# A multi-objective evolutionary algorithm for detecting protein complexes in PPI networks using gene ontology

**DOI:** 10.1038/s41598-025-01667-y

**Published:** 2025-05-15

**Authors:** Mustafa N. Abbas, David Broneske, Gunter Saake

**Affiliations:** 1 Databases and Software Engineering, Otto-von-Guericke-University, Magdeburg, Germany; 2 German Centre for Higher Education Research and Science Studies, Hannover, Germany

**Keywords:** Evolutionary algorithm, Multi-objective optimization, Heuristic perturbation operator, Protein–protein interaction network, Gene ontology, Protein complexes, Computational biology and bioinformatics, Genetics

## Abstract

Detecting protein complexes is crucial in computational biology for understanding cellular mechanisms and facilitating drug discovery. Evolutionary algorithms (EAs) have proven effective in uncovering protein complexes within networks of protein–protein interactions (PPIs). However, their integration with functional insights from gene ontology (GO) annotations remains underexplored. This paper presents two primary contributions: First, it proposes a novel multi-objective optimization model for detecting protein complexes, conceptualizing the task as a problem with inherently conflicting objectives based on biological data. Second, it introduces an innovative gene ontology-based mutation operator, termed the Functional Similarity-Based Protein Translocation Operator ($FS-PTO$). This operator enhances collaboration between the canonical model and the GO-informed mutation strategy, thereby improving the algorithm’s performance. As far as we know, this is the initial effort to incorporate the biological characteristics of PPIs into both the problem formulation and the development of intricate perturbation strategies. We assess the effectiveness of the proposed multi-objective evolutionary algorithm through experiments conducted on two widely recognized PPI networks and two standard complex datasets provided by the Munich Information Center for Protein Sequences (MIPS). To further assess the robustness of our algorithm, we create artificial networks by introducing different noise levels into the original Saccharomyces cerevisiae (yeast) PPI networks. This allows us to evaluate how perturbations in protein interactions affect the algorithm’s performance compared to other approaches. The experimental results highlight that our algorithm outperforms several state-of-the-art methods in accurately identifying protein complexes. Moreover, the findings emphasize the substantial advantages of incorporating our heuristic perturbation operator, which significantly improves the quality of the detected complexes over other evolutionary algorithm-based methods.

## Introduction

Proteins are the cornerstone of all life forms, composed of amino acids linked in polypeptide chains that carry genetic instructions. These molecules are pivotal in performing and regulating the essential functions within organisms through interactions in cellular or controlled environments, as documented in various studies^1–3^. Recent advancements in bioinformatics and biochemistry, particularly in high-throughput techniques such as proteomics, metabolomics, and phenomics, have significantly enhanced our understanding of these processes^4^. Alongside this, the rapid development of computational technologies and high-throughput sequencing methods has empowered researchers to predict potential drug-drug interactions (DDIs), facilitating more accurate and comprehensive analyses of complex biological systems^5,6^. This technological evolution advanced the mapping of protein interactions within intricate biological networks, such as cellular and protein-protein interaction (PPI) networks, with powerful techniques like yeast two-hybrid (Y2H) assays serving as key examples^7,8^.

Despite significant advancements, the study of protein interactions still faces issues such as spurious and missing interactions^9–11^. Often, interactions that exhibit low confidence levels are disregarded in further analyses. Nonetheless, different topological measures and link prediction techniques can successfully detect likely false negatives, enabling the incorporation of highly reliable interactions into PPI networks^12–15^. In biology, it is well-established that proteins with close interactions within PPI networks tend to share functional similarities. Likewise, genes that are under the control of the same transcription factors often exhibit comparable activities and can be associated with similar diseases or phenotypes. This relationship implies that disruptions in these protein interactions may contribute to the development of related diseases or phenotypes^16,17^.

Recently, there has been a significant increase in the literature focusing on various methods for detecting community structure within complex networks. The primary objective of these methodologies is the revelation of hitherto undiscovered structural components within PPI networks. Despite the potential dissimilarities in algorithmic attributes, these techniques generally fall into two main types: heuristic and meta-heuristic approaches, as elucidated by Manipur et al.^18^. Generally, heuristic algorithms are utilized when conventional methods prove insufficient or time-consuming for providing precise solutions. The primary objective of heuristic-based problem-solving is to provide a feasible solution in a timely manner. In contrast, meta-heuristic algorithms are crucial in guiding the search process, often using probabilistic and approximate methods to achieve solutions that are near-optimal. Unfortunately, the computational complexity of the complex detection problem under consideration has been formally established to reside within the realm of nondeterministic polynomial time-hard (NP-hard) complexities, as substantiated by extant literature^19,20^. In combinatorial optimization problems with *n* parameters, exhaustive search for the optimal solution becomes computationally prohibitive as *n* increases. To tackle the complexity of NP-hard problems, meta-heuristic methods, such as evolutionary algorithms (EAs), have been empirically shown to be effective alternatives to traditional heuristics. Additionally, most module identification algorithms focus on detecting densely connected subgraphs, often overlooking smaller or sparsely connected functional modules, which may consist of only two or three proteins^21,22^. To address these challenges, particularly the detection of small or sparse modules and noisy edges, recent algorithms have incorporated prior knowledge, such as co-expression relationships or functional associations. These algorithms improve the network by filtering out low-reliability edges or enhancing it with weighted connections^23^. However, this approach has its limitations. The insufficient integration of domain-specific knowledge can hinder the effectiveness of EAs, as demonstrated by Sala et al.’s study^24^.

To the best of our knowledge, this is the first effort to recast the problem of protein complex identification as a multi-objective optimization (MOO) problem based on biological data. This paper makes two key contributions:

1. Recasting the problem as a multi-objective optimization (MOO) problem: We introduce a novel multi-objective optimization model that integrates both topological and biological data within the evolutionary algorithm framework. This approach accounts for the inherently conflicting effects of intra- and inter-biological properties in PPI networks.
2. Introducing a gene ontology-based mutation operator: We propose a new mutation operator, based on gene ontology (GO), termed the Functional Similarity-Based Protein Translocation Operator ($FS-PTO$), to enhance the consistency and reliability of the results produced by the multi-objective evolutionary algorithm. This operator improves the interaction between topological data and biological insights, ensuring more accurate protein complex identification.

The rest of this paper is structured as follows: Section “Preliminaries” provides an overview of the graph topology and ontology approaches applied to PPI networks. Section “The proposed MOEA-based complex detection algorithm” presents a multi-objective evolutionary algorithm formulated with GO-based methods, focusing on gene ontology and functional data. In Section “Experiments and evaluation”, the results and discussions reveal a strong interest in creating complex detection algorithms that do not rely on ontology-based methods.

## Related works

The methodologies discussed herein span a diverse array of techniques aimed at augmenting local analysis for the characterization of protein complexes within PPI networks, predominantly focusing on network density.

Dongen et al.^25^ proposed the Markov Cluster (MCL) algorithm, which is intended to simulate the behavior of a random walk on a graph. This algorithm effectively captures protein families by utilizing two key operations: expansion and inflation. Expansion allows the random walk to spread across the graph, while inflation sharpens the clusters by favoring stronger connections and suppressing weaker ones. Due to these operations, the MCL algorithm is highly regarded for its ability to accurately cluster graphs, and it has been widely recognized as one of the most effective techniques for this purpose^26^.

In a different approach, Bader and Hogue^27^ presented the Molecular Complex Detection (MCODE) algorithm in their study, which serves as a computational tool for identifying protein complexes in large-scale protein interaction networks. MCODE algorithm operates on a graph-growing principle, employing a greedy strategy to assemble clusters of proteins centered around a selected seed vertex. The process begins by choosing a single protein as the seed vertex. Subsequently, the algorithm evaluates neighboring proteins in the network, adding them to the forming cluster if their pre-computed weights are sufficiently similar to that of the seed vertex, based on a predetermined threshold. This iterative inclusion continues until no additional proteins meet the criteria for inclusion. Through this methodical approach, MCODE effectively identifies densely interconnected regions within the network, which are indicative of potential protein complexes.

Expanding on network analysis techniques, Li et al. ^28^ presented the DECAFF (Dense-Neighborhood Extraction using Connectivity and Confidence Features) algorithm, marking a significant improvement in network analysis. DECAFF stands out due to its unique approach, which integrates a method for removing hubs with a technique for combining local cliques. Central to the algorithm is a probabilistic model specifically designed to evaluate the reliability of connections within complex networks. This model effectively filters out unreliable or spurious connections, thereby enhancing the precision of the analysis. The hub-removal strategy is a particularly critical component of DECAFF, as it addresses a major challenge in network analysis: the presence of highly connected nodes, or hubs, which can obscure the detection of meaningful community structures. By systematically removing these hubs, the algorithm reduces noise in the network, facilitating the clearer identification of densely connected subgraphs, or cliques.

Zaki et al.  ^29^ proposed a novel approach to improve the identification of protein complexes using graph convolutional network (GCN) techniques. Their method starts by redefining the problem as a node classification task, where the goal is to detect protein complexes within a graph. In this framework, each protein is represented as a node, and the objective is to classify these nodes into distinct complex groups. Following this redefinition, the authors develop a sophisticated model tailored for this classification task. A central element of their approach is the creation of a detailed complex affiliation matrix. This matrix is crucial for organizing and grouping the nodes, which represent individual proteins, thus enabling a more structured method for identifying complex formations. To improve the extraction of relevant features from the nodes, the authors utilize an advanced GCN feature extractor. This tool is essential for capturing the intricate characteristics of each node, which are critical for precise classification. Additionally, they employ a mean shift clustering algorithm, which further refines the grouping of nodes based on the features extracted by the GCN. This clustering technique enhances the identification and delineation of protein complexes by grouping proteins with similar features.

Accurately detecting protein complexes within PPI networks presents a significant challenge, as conventional methods often prove inadequate due to their complexity and the limitations of traditional computational approaches. This problem, classified as NP-hard, makes it extremely difficult to find precise solutions using standard techniques. To address this, researchers have increasingly turned to optimization algorithms inspired by natural processes. Among these, genetic algorithms have gained particular attention. Various frameworks based on genetic algorithms have been developed to explore complex problem spaces and find solutions that are near-optimal. These nature-inspired techniques utilize evolutionary principles to iteratively refine solutions, making them well-suited to address the specific challenges posed by the complexities of PPI networks^30^.

Pizzuti and Rombo^31,32^ tackled the challenge of identifying protein complexes within PPI networks through the optimization of single-objective models. They introduced a range of quality functions to serve as fitness measures in their optimization framework. These metrics include Modularity (Q), which assesses the network’s division into modules; Conductance (CO), which evaluates the share of edges that link a cluster to the remainder of the network; Expansion (EX), measuring how a cluster extends beyond its core; Cut Ratio (CR), focusing on the ratio of edges cut relative to the total number of edges; Normalized Cut (NC), which normalizes the cut criterion based on network size; Internal Density (ID), quantifying the density of connections within a cluster; and Community Score (CS), a composite measure of cluster quality. By employing these metrics, Pizzuti and Rombo significantly advanced the identification of hidden protein complexes, thereby enhancing our understanding of protein interactions and functions.

Building on this, Cao et al.^33^ proposed an innovative multi-objective algorithm, MOEPGA, which further refines the analysis of PPI networks by considering multiple topological features. Unlike the single-objective models, MOEPGA incorporates network size, characteristic path length (CPL), and density into its optimization process. The MOEPGA algorithm follows a systematic approach, beginning with an in-depth analysis of the PPI network to extract relevant topological properties. These properties are then utilized to formulate a comprehensive multi-objective function that guides the optimization process. The algorithm operates in a structured manner, where each subgraph undergoes three fundamental steps: population initialization, mutation, and selection. Population initialization ensures a diverse starting set of solutions, mutation introduces variations to explore different network configurations, and selection refines the solutions by preserving the most optimal subgraphs. By integrating these steps, the MOEPGA algorithm enhances the identification of significant network structures and contributes to a more effective analysis of complex biological networks.

In a similar vein, Vella et al.^34^ propose MTGO (Module detection via Topological information and GO knowledge), a method that combines both topological and functional insights for module detection. This approach goes a step further by integrating Gene Ontology (GO) terms during module construction, assigning the most appropriate GO term to each module, and thus enhancing functional interpretation. By repeatedly partitioning the network, MTGO refines module structures based on both GO annotations and graph modularity, creating a more comprehensive and biologically meaningful framework for understanding protein-protein interactions.

Extending the work of previous methods, Bandyopadhyay et al.^35^ incorporated both biological and topological properties into a multi-objective optimization framework aimed at identifying protein complexes and determining their disease associations. This method introduces a more integrated approach, combining structural and biological features to optimize the identification of protein complexes. The optimization problem is defined through three objective functions: two focusing on topological properties and one addressing biological aspects. The first topological property is formalized as an objective function that seeks to maximize the contribution of a node in the protein interaction network. The contribution of a node $n_i$ within a protein cluster *C* is defined as follows:

1 $$\begin{aligned} \max Contr(n_i) = \sum _{n_i \in C} \frac{|N_{n_i}|}{degree(n_i)} \end{aligned}$$

where $N_{n_i}$ denotes the set of nodes directly connected to node $n_i$ in cluster *C*, and $degree(n_i)$ represents the degree of the node. The degree of a node, $degree(n_i)$, refers to the number of edges connected to the node, which quantifies its immediate connectivity within the network. The term $|N_{n_i}|$ refers to the size of the neighborhood of node $n_i$, or the count of nodes directly adjacent to $n_i$ in cluster *C*. The goal of this function is to generate compact and well-separated clusters by favoring nodes with fewer external connections, thereby minimizing interaction partners outside the cluster. In essence, this objective function prioritizes nodes that contribute more locally to the cluster and reduces the influence of external nodes, leading to more cohesive protein clusters.

The second topological property focuses on the concept of closeness centrality, which is used to measure how efficiently a node spreads information across the network. Closeness centrality is defined as the reciprocal of the average shortest-path distance from a given node to all other nodes in the graph. A higher closeness centrality value indicates that a node is more centrally positioned within the network, which is a key factor in forming protein complexes. The corresponding objective function, which seeks to maximize the closeness centrality of nodes in a protein complex, is expressed as:

2 $$\begin{aligned} \max \sum _{n_i \in C} CC(n_i) \end{aligned}$$

where $CC(n_i)$ denotes the closeness centrality of node $n_i$ within the protein cluster *C*, and the sum is taken over all nodes that belong to the protein complex *C*. The optimization goal is to maximize the total closeness centrality for all nodes in the cluster, ensuring that the resulting protein complexes consist of nodes that are centrally located in the network. This centrality promotes the identification of biologically significant protein complexes, as nodes with higher closeness centrality are typically more important in the overall structure of the protein interaction network. By prioritizing these central nodes, the algorithm can enhance the biological relevance of the identified complexes, improving the accuracy of protein complex detections.

The third objective function aims to ensure that proteins within the identified protein complexes are functionally similar. This is achieved by computing the semantic similarity between the GO terms with which the proteins are annotated. Since each protein is annotated with multiple GO terms, the semantic similarity between two proteins is calculated by averaging the similarities of all cross-pairs of GO terms associated with them^36^.

The semantic similarities between all protein pairs in the PPI network are precomputed and assigned as edge weights in a semantic similarity network. The objective is to maximize the average similarity of all protein pairs connected by edges in a chromosome, which represents a potential protein complex. This can be formulated as the following objective function:

3 $$\begin{aligned} \max \frac{1}{|E|} \sum _{(n_i, n_j) \in E} Sim(n_i, n_j) \end{aligned}$$

where *E* is the set of edges in the semantic similarity network, $(n_i, n_j)$ is a pair of connected proteins, and $Sim(n_i, n_j)$ represents the semantic similarity between proteins $n_i$ and $n_j$. By maximizing the average semantic similarity of protein pairs in a chromosome, this objective ensures that the identified protein complexes are functionally coherent, reflecting shared biological functions within each complex.

In the latest advancements, Abbas et al. ^37^ have recently introduced a heuristic mutation operator known as strong neighbor-node migration. This operator is designed to improve the performance of optimization algorithms, whether they address a single objective or multiple objectives. This innovative operator improves the quality of solutions produced by these algorithms, significantly advancing their performance and effectiveness in solving complex optimization problems related to protein complex detection.

## Preliminaries

**Fig. 1:**
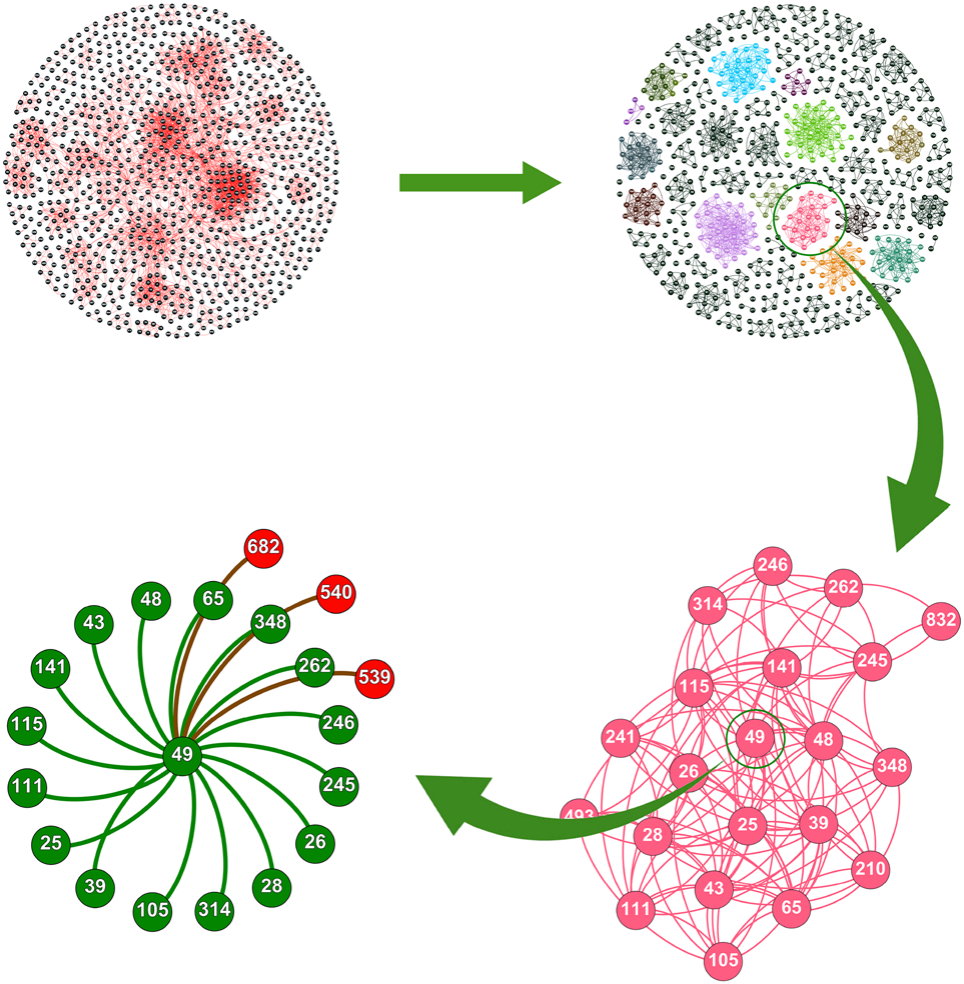
A comprehensive visualization of the yeast Saccharomyces cerevisiae protein network (Yeast-D1), comprising 990 proteins and 4687 interactions. The figure illustrates the segmentation into 81 complexes (top right), provides a detailed view of a specific complex (bottom right), and focuses on protein #49 (’YBR198C’) to display its internal and external connections (bottom left).

Understanding the complex network of cellular processes starts with examining the PPI network. This complex web of interactions can be modeled as a graph $G(\mathbb {V}, \mathbb {E})$, where $\mathbb {V}$ represents the *n* vertices, $\mathbb {V} = \{v_1, v_2, \dots , v_n\}$, each corresponding to a protein. The edges $\mathbb {E}$ are pairs $(v_i, v_j)$ signifying interactions between proteins $P_i$ and $P_j$. The degree $d_i$ of a protein $P_i$ quantifies the number of interactions involving $P_i$, defined mathematically as $d_i = \left| { (v_i, v_j) \in \mathbb {E} } \right|$. Figure 1 presents a detailed visualization of the yeast Saccharomyces cerevisiae (Yeast-D1). The top left section shows a comprehensive network of 990 proteins and 4,687 interactions. This network is organized into 81 distinct complexes, according to benchmark datasets from the Munich Information Center for Protein Sequence (MIPS) catalog^38^. The top right section illustrates these complexes, which vary in size and offer a detailed view of the network’s structure. The bottom right section zooms in on one specific complex, which contains 21 proteins connected through multiple intra-complex interactions, highlighting their functional relationships. In the bottom left section, protein #49 (’YBR198C’) is emphasized within its complex, with its internal interactions shown in green. Additionally, its interactions with proteins #682, #540, and #539 from a different complex are highlighted in red, demonstrating its connections across various molecular groups.

**Fig. 2:**
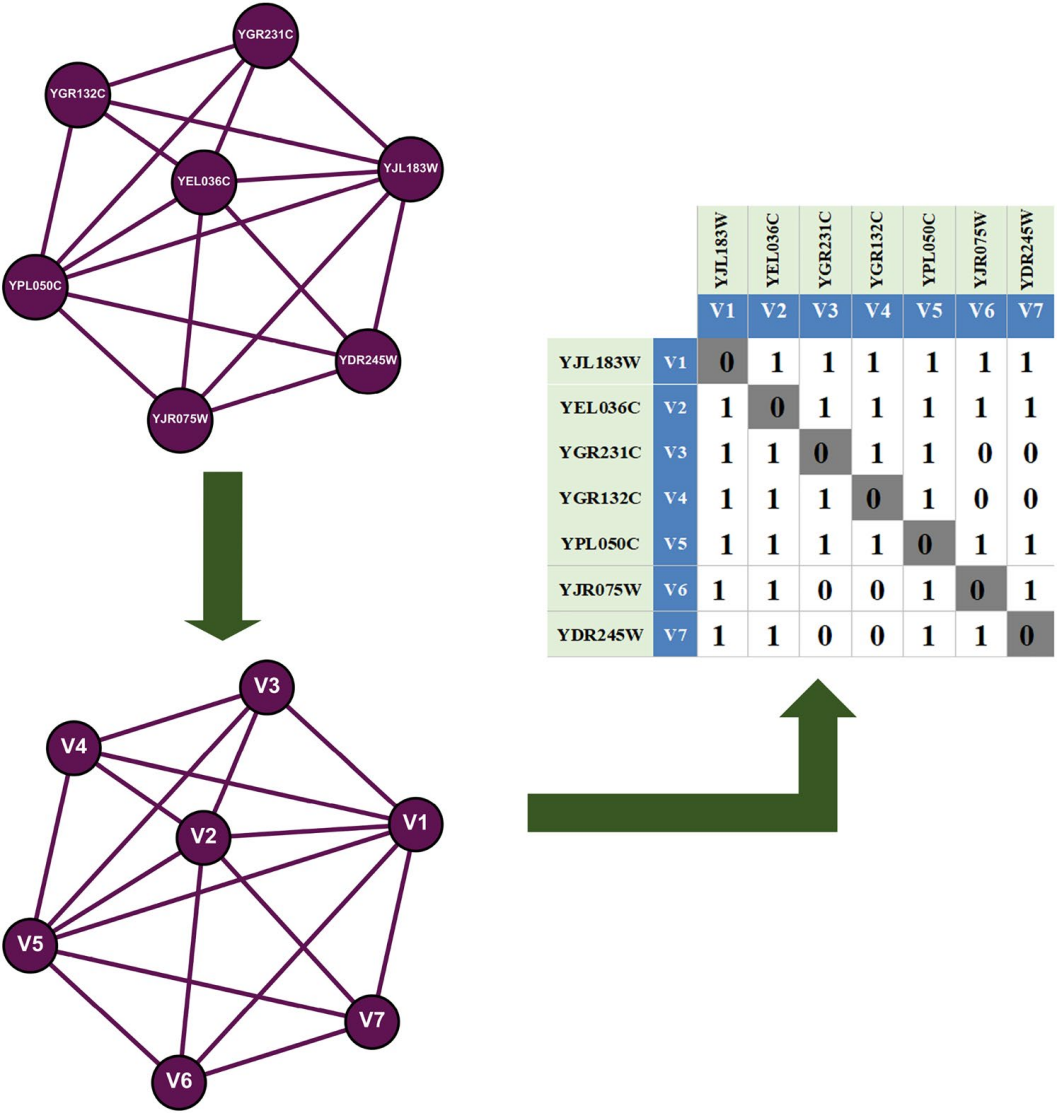
Illustration of seven proteins in Saccharomyces cerevisiae along with their corresponding adjacency matrix.

In undirected graphs such as the PPI network, the structure is commonly depicted using a symmetric adjacency matrix, denoted by $\textbf{A} = [a_{ij}]^{n \times n}$. This matrix outlines the connections between nodes, where each element $a_{ij}$ indicates whether a direct interaction exists between the proteins $P_i$ and $P_j$. Specifically, if $a_{ij} = 1$ and $a_{ji} = 1$, it denotes the presence of an interaction, while $a_{ij} = 0$ and $a_{ji} = 0$ indicate no interaction. In matrix $\textbf{A}$, each row and column represent a particular protein node, highlighting direct interactions. Figure 2 provides an example of such an adjacency matrix, illustrating a segment of a PPI network.

To further analyze the PPI network, we explore the space of possible decompositions of *G* into complexes, denoted as $\Omega$. This space includes all clustering solutions derived from decomposing the adjacency matrix $\textbf{A}$. The adjacency matrix $\textbf{A}$ encodes the interactions between a defined set of proteins, denoted as $\mathcal {P} = {P_1, P_2, \dots , P_N}$. By analyzing $\textbf{A}$, $\Omega$ identifies all feasible partitions of $\textbf{A}$ into unequal square sub-matrices, representing potential complexes. For a specific clustering solution $\mathcal {C} \in \Omega$, where $\mathcal {C} = {C_1, C_2, \dots , C_K}$ is a decomposition of *G* into *K* complexes, we can quantify the connectivity patterns of a protein $P_i \in \mathcal {P}$. Specifically, for a complex $C_i \in \mathcal {C}$, the intra-complex degree and inter-complex degree of $P_i$ are calculated using the following formulas:

4 $$\begin{aligned} d_{i, \text {intra}}= & \sum _{P_j \in C_i} a_{ij} \end{aligned}$$

5 $$\begin{aligned} d_{i, \text {inter}}= & \sum _{P_j \notin C_i} a_{ij} \end{aligned}$$

### Gene ontology: exploring semantic and functional similarity

**Fig. 3:**
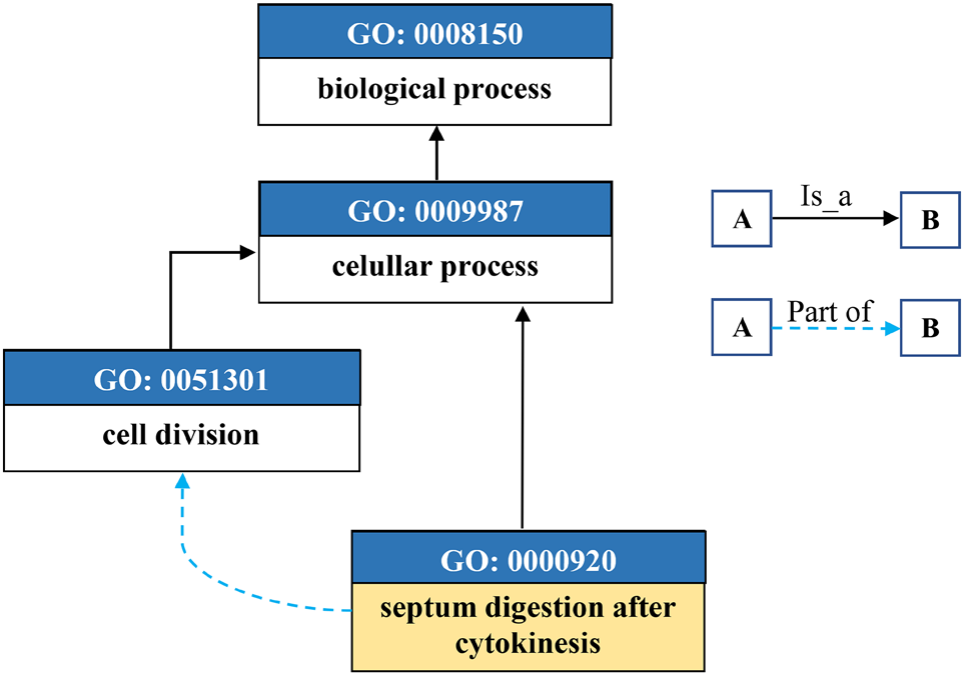
DAG representation of the GO term Septum Digestion After Cytokinesis (GO:0000920), highlighting its relationships with other GO terms. The solid arrow represents the ‘$is\_a$’ relationship, while the dotted arrow indicates the ‘$part\_of$’ relationship.

To fully understand the complex roles of genes and their products in various biological contexts, it is essential to adopt a structured approach for describing their functions. The Gene Ontology (GO) framework provides a robust and widely accepted system for this purpose. GO is a comprehensive, collaboratively curated public database that standardizes the characterization of gene products using a controlled vocabulary, allowing for consistent and thorough descriptions of their roles within cellular contexts. This system is organized into three primary ontologies: biological process (*BP*), cellular component (*CC*), and molecular function (*MF*), each addressing distinct aspects of gene product activities. These ontologies are represented as directed acyclic graphs (DAGs). To further enrich the understanding of gene functions, the assignment of a gene product to specific terms within these ontologies is referred to as a Gene Ontology annotation (*GOA*)^39^. A gene product, denoted as $P_i$, is typically annotated with a set of terms known as GO Slim terms, $\mathcal {T}_{P_i}$, which provide a concise summary of its functional attributes. This relationship is represented as follows:

6 $$\begin{aligned} \mathcal {T}_{P_i} = \bigcup _{x \in \{MF, BP, CC\}} x_i \end{aligned}$$

here, $\mathcal {T}_{P_i}$ represents the set of GO Slim terms associated with gene product $P_i$, and the union over the ontologies captures the functional attributes of $P_i$ across all three domains: *MF*, *BP*, and *CC*. The DAGs represent semantic relationships between terms through two primary types of edges: ‘$is\_a$’ and ‘$part\_of$. The ‘$is\_a$’ relationship denotes hierarchical classification, indicating that one term is a subclass of another. For instance, if term *A* is categorized as an ‘$is\_a$’ instance of term *B*, *A* is understood to be a more specific version of *B*, inheriting all attributes of *B* while adding its own unique characteristics. In contrast, the ‘$part\_of$’ relationship illustrates a componential or structural connection, where a term *C* is described as ‘$part\_of$’ another term *D*, meaning that *C* is a constituent of *D* whenever *C* is present, though its presence may vary depending on specific conditions within the biological system^40^. To understand the semantic similarity between GO terms, it is crucial to convert their semantics into a numerical format. This approach allows us to evaluate how biologically related different GO terms are based on their positions and connections within the GO graph. By representing the terms through a DAG, which maps the term’s path to the root terms, we can effectively capture these relationships. For example, Figure 3 illustrates the DAG for the GO term Septum Digestion After Cytokinesis (0000920). This graph displays how the term is related to others through different types of connections: a solid arrow indicates an ‘$is\_a$’ relationship, showing that Septum Digestion After Cytokinesis is a subclass of Cellular Process (0009987), while a dotted arrow signifies a ‘$part\_of$’ relationship, highlighting its role as a component of Cell Division (0051301).

**Fig. 4:**
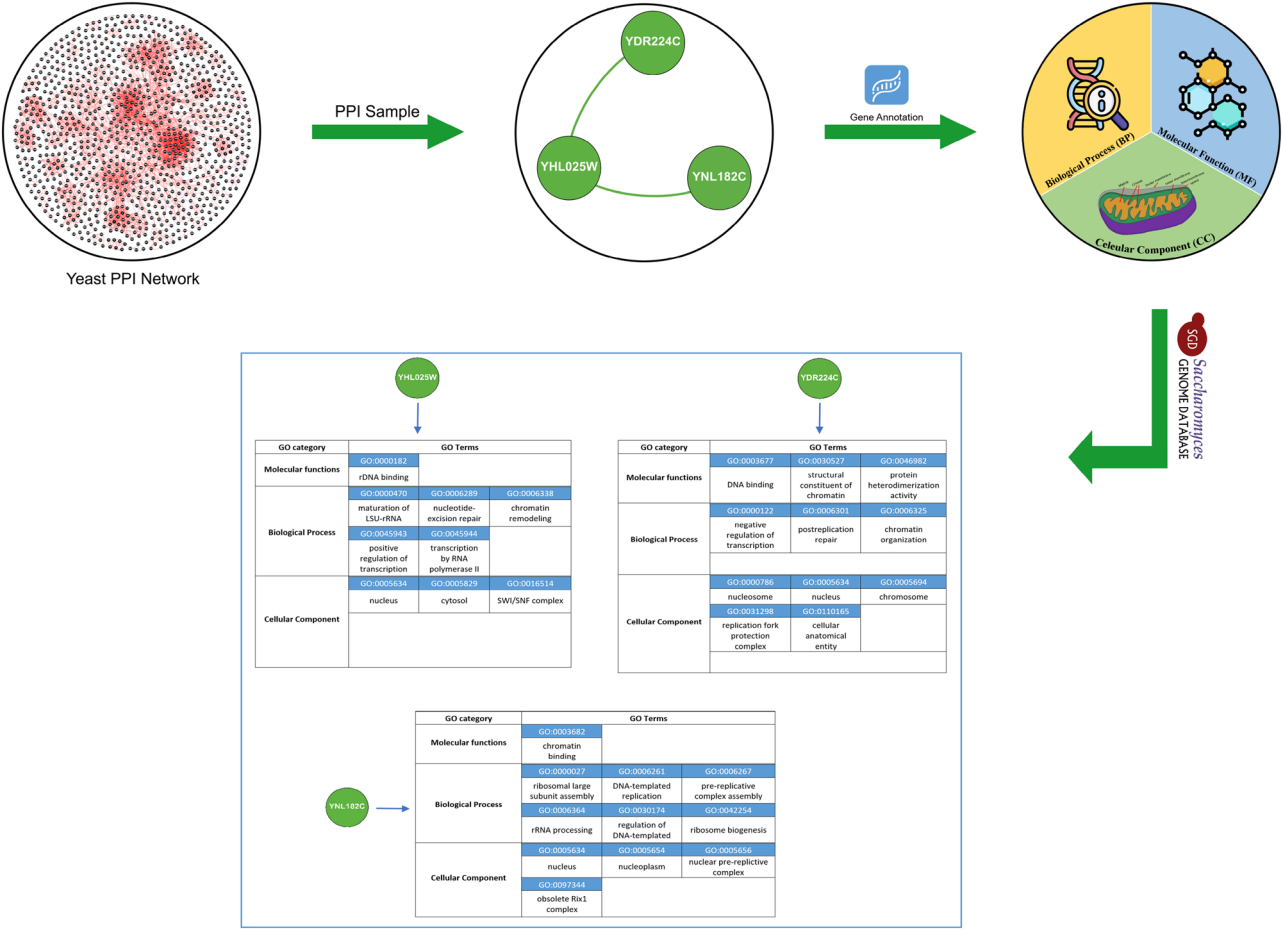
Annotation of three distinct protein from the yeast PPI network with their respective GO terms.

Proteins can be associated with multiple GO terms, reflecting the diversity of their biological roles and functions. However, some proteins may remain unannotated due to limitations in GO data and the broad range of protein functions. For example, Fig. 4 presents the GO annotations for three proteins from the yeast PPI network, highlighting their associated biological processes, molecular functions, and cellular components. This detailed information is sourced from the most recent comprehensive datasets available in the Saccharomyces Genome Database (SGD), which can be further explored at http://www.yeastgenome.org.

#### Semantic similarity of GO terms

Semantic similarity, represented as *SS*, is a crucial metric for assessing the relatedness or similarity among GO terms. It achieves this by considering both their hierarchical relationships and the meaning conveyed through their annotations. Its significance lies in its ability to facilitate comparisons among sets of genes and the discovery of functional relationships among genes^41–43^. To compute semantic similarity, a semantic similarity matrix is constructed and denoted as $\textbf{S}=[SS_{ij}]^{N\times N}$, where $SS_{ij}$ represents the semantic similarity between terms *A* and *B*, while *N* represents the number of GO terms used to annotate a set of *n* proteins. Semantic similarity between GO terms can be explored through two main strategies: internal methods, which analyze the inherent structure of the GO DAGs, and external methods, which rely on external data sources like annotation corpora. Internal methods exclusively utilize the ontological topology to evaluate relationships, offering a focused perspective on the semantic connections within the DAG structure. A prominent internal method is the hybrid approach, which integrates aspects of both path length metrics and structural properties of the DAG. By combining these elements, the hybrid model provides a more refined and nuanced evaluation of semantic similarity. Building upon this foundation, the hybrid approach determines edge weights through two principal factors: node density, reflecting the number of shared and unique ancestors between terms, and link type, such as ‘$is\_a$’ or ‘$part\_of$’. These weighted edges are then applied to compute the semantic similarity, making the hybrid model an effective framework for analyzing relationships between GO terms. Unlike earlier models, such as the one proposed by Wang et al.^40^, which included the root of the ontology in calculating semantic values, the hybrid approach focuses on a more nuanced method inspired by Kamran et al.^44^. Kamran’s method, GOntoSim, enhances the calculation of semantic similarity by considering the graph structure and the information content of the nodes, while accurately capturing the similarity between the ancestors of GO terms and accounting for their common children. By excluding the root from the calculation, this approach provides a more precise reflection of the inherent semantic relationships between terms. The semantic contribution of a GO term from its higher-level DAG is represented as $DAG_A = (A, T_A, E_A)$, where ($T_A$) includes the set of GO terms related to a specific GO term (*A*) and its ancestors, and ($E_A$) denotes the set of edges connecting these nodes within $DAG_A$. Each edge in $E_A$ is assigned a weight ($W_e$) that reflects the type of relationship it represents in the graph. For this analysis, $W_e$ values are set to 0.8 for the ‘$is\_a$’ relationship, 0.6 for the ‘$part\_of$’ relationship, and 0.7 for the ‘regulates’ relationship. In evaluating the semantic contribution for $DAG_A$, the GO term, *A*, is given a maximum contribution value of 1. For all other terms in the DAG, the contribution of an ancestor term ($t^\prime$) relative to *A* is calculated as the highest product of weights along the path from *A* to ($t^\prime$). The root of the ontology is assigned a semantic contribution value of zero for the term *A*.

7 $$\begin{aligned} \begin{aligned}&S_A(t) = {\left\{ \begin{array}{ll} S_A(root) = 0 \\ S_A(A) = 1 \\ \max \{W_e \times S_A(t^\prime )\} \vert t^\prime \in \text {children of } (t), if t \ne A \end{array}\right. } \end{aligned} \end{aligned}$$

The cumulative semantic value *SV*(*A*) of a GO term *A* is determined by aggregating the semantic contributions of the term along with those of its ancestor terms.

8 $$\begin{aligned} SV(A) = \sum _{t \in T_A} S_A(t) \end{aligned}$$

The semantic similarity *SS*(*A*, *B*) between two GO terms *A* and *B* is calculated by taking the sum of the semantic contributions of intersecting terms *A* and *B*, divided by the sum of the total semantic values of *A* and *B*.

9 $$\begin{aligned} SS(A, B) = \frac{\sum _{t \in T_A \cap T_B}{(S_A(t)) + S_B(t))}}{SV(A) + SV(B)} \end{aligned}$$

#### Functional similarity

Gene product similarity (*FS*) is used to compare the functional similarity between genes or proteins based on their annotations. This similarity is typically evaluated using two main approaches: group-wise and pairwise^45^.

The group-wise approach considers the collective properties of annotation sets and is further categorized into three subtypes: set-based, graph-based, and vector-based methods. Set-based approaches leverage traditional cardinality-based measures like the Jaccard index, which evaluates the ratio of shared terms to the total terms, and the Dice coefficient, which emphasizes shared terms relative to the average size of the annotation sets. These methods are straightforward but may oversimplify relationships between terms. Graph-based methods, on the other hand, exploit the hierarchical structure of ontologies, to capture the relationships between terms. In contrast, the pairwise approach evaluates *FS* by directly comparing the terms associated with two proteins, $P_1$ and $P_2$, using their respective sets of annotations, $\mathcal {T}_{P_1}$ and $\mathcal {T}_{P_2}$. This method calculates the semantic similarity (*SS*) between each pair of terms, either by considering all possible term pairs or by focusing on the best-matching pairs. The *SS* values are then combined into a single functional similarity score for the two proteins. Different statistical methods, such as averaging, summing, or taking the maximum or minimum similarity scores, can be used to derive the global *FS*. A widely used measure for *FS* is the maximum similarity, defined as:

10 $$\begin{aligned} FS(P_1, P_2) = \underset{A \in \mathcal {T}_{P_1}, \, B \in \mathcal {T}_{P_2}}{\text {argmax}} \, SS(A, B). \end{aligned}$$

## The proposed MOEA-based complex detection algorithm

The need to address multiple conflicting objectives simultaneously is a common challenge in many real-world problems, driving the motivation for MOO. Researchers have increasingly focused on this area due to its ability to capture the complexity of such problems more effectively than traditional single-objective approaches. By leveraging MOO, it becomes possible to identify a set of Pareto-optimal solutions rather than a single optimal or near-optimal solution. This approach provides decision-makers with a spectrum of non-dominated solutions, each representing an optimal or near-optimal trade-off among the conflicting objectives, thereby facilitating more informed and balanced decision-making^46^. Building on the motivation for multi-objective optimization, we introduce a new model for detecting protein complexes within the context of large-scale PPI networks. Recognizing the complexity and scale of these networks, our model leverages the decomposition-based multi-objective evolutionary algorithm (MOEA/D) developed by Zhang and Li^47^, which is well-suited for handling multiple conflicting objectives simultaneously. We have specifically adapted the core structure of MOEA/D to better accommodate the unique challenges posed by PPI networks.

Our model integrates both topological and biological characteristics of protein complexes, ensuring a comprehensive optimization process that balances these conflicting aspects. To further enhance the model’s effectiveness, we introduce a heuristic perturbation operator that exploits biological features, resulting in more precise and reliable detection of protein complexes within extensive PPI networks.

### Objective functions

The proposed MOEA framework seeks to bridge the gap between evolutionary algorithms and principles observed in biological systems. Beyond the existing topological domain $\textbf{A}$, we introduce two novel domains: semantic similarity of gene ontology (denoted as $\textbf{SS}$) and similarity of protein functions (denoted as $\textbf{FS}$). By integrating these additional domains, the framework is poised to advance the effectiveness of evolutionary algorithms in identifying protein complexes.

In this study, we adopted a variant of our methodology designed to identify functional similarity by conducting a pairwise analysis of direct terms associated with protein pairs, using the Best Match Average (BMA) method. In this approach, each term linked to the first protein is paired with its closest counterpart in the second protein, and vice versa. This process constructs a functional similarity matrix, denoted as $\textbf{FS}_{BMA} = [FS_{ij}]^{n \times n}$, where $FS_{ij}$ represents the functional similarity between the direct GO terms of protein pair $P_i$ and $P_j$. The functional similarity is calculated using the following equation:

11 $$\begin{aligned} FS_{BMA}(P_1, P_2) = \frac{\sum _{A \in \mathcal {T}_{P_1}} \max _{B \in \mathcal {T}_{P_2}} SS(A, B) + \sum _{B \in \mathcal {T}_{P_2}} \max _{A \in \mathcal {T}_{P_1}} SS(A, B)}{|\mathcal {T}_{P_1}| + |\mathcal {T}_{P_2}|} \end{aligned}$$

To further refine the semantic analysis, we employed the GOntoSim method introduced by^44^, which evaluate the similarity between GO terms and their corresponding DAGs. This method produces a semantic similarity matrix, represented as:

12 $$\begin{aligned} \textbf{S}_{\text {GOntoSim}} = [SS_{ij}]^{N \times N}, \end{aligned}$$

where each element $SS_{ij}$ represents the semantic similarity between the DAG terms $A_i$ and $B_j$, computed using GOntoSim. Formally, the semantic similarity between two terms $T_A$ and $T_B$ can be expressed as:

13 $$\begin{aligned} SS_{ij} = \text {GOntoSim}(DAG(T_A), DAG(T_B)), \end{aligned}$$

where $DAG(T_A)$ and $DAG(T_B)$ denote the respective DAG structures of the terms $T_A$ and $T_B$. The GOntoSim method leverages the structural and hierarchical relationships in the DAGs, including ancestor terms and edge weights, to calculate a numerical similarity score. The resulting matrix $\textbf{S}_{\text {GOntoSim}}$ serves as the foundation for downstream analysis of GO term relationships.

While these methods enhance the precision of functional and semantic similarity assessments, a critical limitation remains in the existing models. For instance, the work by Bandyopadhyay et al.^35^ focuses on optimizing non-conflicting, predominantly topological objectives in their MOO model. By simplifying the trade-offs and narrowing the solution space, this approach limits the discovery of diverse protein complexes. Furthermore, the absence of conflicting objectives overlooks the biological trade-offs inherent in real-world protein interactions.

To address these limitations, our formulation introduces two biologically conflicting objectives, focusing on both intra-biological and inter-biological properties of protein complexes. By incorporating these conflicting objectives, we aim to generate a set of near Pareto-optimal solutions, where improvements in one objective cannot be achieved without a corresponding trade-off in the other. This allows for a more biologically relevant exploration of the solution space, capturing the inherent complexity of protein interactions. To further refine the complex detection process, the proposed multi-objective optimization model refines the approach by narrowing its focus to two fundamental optimization functions. These functions are the Intra-Complex Semantic ($\text {ICS}_{\text {Intra}}$) score, which evaluates semantic consistency within a given complex, and the Inter-Complex Semantic ($\text {ICS}_{\text {Inter}}$) score, which assesses the semantic relationships between different complexes. These functions are designed to assess the effectiveness of a solution in terms of functional similarity among complexes. Each complex, denoted as ($C_k$), is evaluated using three biological attributes. The first attribute includes general semantic features, such as the semantic volume ($V_k$) and the size ($|C_k|$) of the complex. The second attribute focuses on the contributions from proteins with high semantic similarity within the complex, specifically the relative input these proteins ($R_k$). The third attribute is the cohesiveness or semantic centrality of the complex, quantified by the semantic centrality measure ($D_k$). To achieve a balanced optimization, we aim to maximize the term ($\frac{(V_k + R_k)}{|C_k|}$) while minimizing the cohesiveness measure ($D_k$). To unify these objectives into a single minimization framework, we modify the term ($\frac{(V_k + R_k)}{|C_k|}$) by subtracting it from ($|C_k|^2$). Consequently, the $\text {ICS}_{\text {Intra}}$ score for a partition solution ($\mathcal {C} = {C_1, C_2, \dots , C_K}$) is expressed as follows:

14 $$\begin{aligned} \min \text {ICS}_{\text {Intra}}(\mathcal {C}) = \sum _{k=1}^K \left( \frac{|C_k|^2 - (V_k + R_k)}{|C_k|} + D_k\right) \end{aligned}$$

where $R_k$ quantifies the contribution of proteins with high semantic similarity within $C_k$ and is computed as:

15 $$\begin{aligned} R_k = \sum _{v \in C_k \mid \text {IS}_{\text {Intra},k}(v) > \text {IS}_{\text {Inter},k}(v)} \frac{\text {IS}_{\text {Intra},k}(v)}{\text {IS}_{\text {Intra},k}(v) + \text {IS}_{\text {Inter},k}(v)} \end{aligned}$$

here, $\text {IS}_{\text {Intra},k}(v)$ represents the intra-complex semantic similarity of protein *v* within complex $C_k$, while $\text {IS}_{\text {Inter},k}(v)$ represents the inter-complex semantic similarity of protein *v* with proteins in other complexes.

Additionally, the cohesiveness measure $D_k$ of complex $C_k$ is defined by:

16 $$\begin{aligned} D_k = \frac{\sum _{v,w \in C_k} \text {sem\_dist}(v,w)}{|C_k|} \end{aligned}$$

A lower $\text {ICS}_{\text {Intra}}$ score indicates that the complexes are more compact, semantically coherent, and contain a higher proportion of proteins with high semantic similarity.

On the other hand, the $\text {ICS}_{\text {Inter}}$ score is essential for assessing the separation between complexes. This score aggregates the total inter-complex protein semantic similarity $\left( \sum _{v \in C_i} \text {IS}_{\text {Inter},i}(v)\right)$ and the number of proteins exhibiting stronger semantic similarity to proteins in other complexes than to those within the same complex ($\text {weak}_i$). The $\text {ICS}_{\text {Inter}}$ score for a partition solution ($\mathcal {C}$) is defined as:

17 $$\begin{aligned} \min \text {ICS}_{\text {Inter}}(\mathcal {C}) = K \cdot \sum _{i=1}^K \left( \frac{\sum _{v \in C_i} \frac{\text {IS}_{\text {Inter},i}(v)}{\text {IS}_{\text {Intra},i}(v)}}{|C_i|} + weak_i \right) \end{aligned}$$

In this formula, $\text {weak}_i$ represents how many proteins within the complex $C_i$ have an inter-complex semantic similarity score that surpasses their intra-complex similarity score. For example, if complex $C_i$ contains proteins $P_1$, $P_2$, and $P_3$, with the following similarity scores:

- For protein $P_1$: $\text {IS}_{\text {Intra},i}(P_1) = 0.4$ and $\text {IS}_{\text {Inter},i}(P_1) = 0.7$
- For protein $P_2$: $\text {IS}_{\text {Intra},i}(P_2) = 0.5$ and $\text {IS}_{\text {Inter},i}(P_2) = 0.6$
- For protein $P_3$: $\text {IS}_{\text {Intra},i}(P_3) = 0.6$ and $\text {IS}_{\text {Inter},i}(P_3) = 0.5$

Here, proteins $P_1$ and $P_2$ are considered *weak* because their inter-complex similarities (0.7 and 0.6) exceed their intra-complex similarities (0.4 and 0.5). Thus, $\text {weak}_i$ would be 2 for this complex.

The effectiveness of $\text {ICS}_{\text {Inter}}$ in maintaining distinct protein complexes relies on accurately quantifying semantic similarity. Semantic similarity serves as a foundational measure for evaluating the relatedness of GO terms based on their hierarchical positions within the GO DAG. In this study, we employed GOntoSim^44^, a recent method that leverages these hierarchical relationships to quantify term similarity, providing a biologically meaningful representation of functional associations. Extending this concept to gene products, functional similarity aggregates the semantic similarity of their associated GO terms. The BMA method, employed in our study, refines functional similarity computation by pairing each term from one protein with its most similar counterpart in another protein. This process results in a comprehensive similarity matrix, which plays a crucial role in assessing protein complex formation. By integrating semantic and functional similarity measures into our multi-objective optimization model, we introduce biologically relevant trade-offs that refine protein complex detection. Specifically, the Intra-Complex Semantic Score ($\text {ICS}_{\text {Intra}}$) ensures that proteins within the same complex exhibit high functional coherence, while the Inter-Complex Semantic Score ($\text {ICS}_{\text {Inter}}$) penalizes excessive similarity between proteins assigned to different complexes. These two objectives inherently conflict, as maximizing intra-complex cohesion often increases inter-complex similarity.

### Chromosome representation

In a population, $\mathbb {I}$, each solution, referred to as a chromosome *I*, consists of *n* genes associated with proteins in the PPI network. Each gene features a locus and an allele value: the locus *i* identifies a specific protein $P_i$, while the allele value *j* denotes an interacting neighboring protein of $P_i$. Thus, each gene represents a potential interaction between two proteins. The encoding scheme for the genotype ensures the generation of feasible solutions, preventing invalid configurations such as disconnected nodes or erroneous interactions.

Mathematically, a chromosome is represented as:

18 $$\begin{aligned} I = \{ I_1, I_2, \dots , I_n \} \end{aligned}$$

where each gene $I_i$ is described by:

19 $$\begin{aligned} I_i = (P_i, j) \quad \text {with } j \in \mathbb {N}(P_i) \end{aligned}$$

here, $\mathbb {N}(P_i)$ denotes the set of neighboring proteins that interact with protein $P_i$. The decoding function $\gamma$ applied to a chromosome *I* yields a set of possible protein complexes. This function is given by:

20 $$\begin{aligned} \gamma (I) = \mathcal {C} = \{ C_1, C_2, \dots , C_K \} \end{aligned}$$

where $\mathcal {C}$ represents the collection of protein complexes formed by the proteins encoded in the chromosome, and *K* is the number of distinct complexes, which may differ among chromosomes.

### Recombination operator

**Fig. 5:**
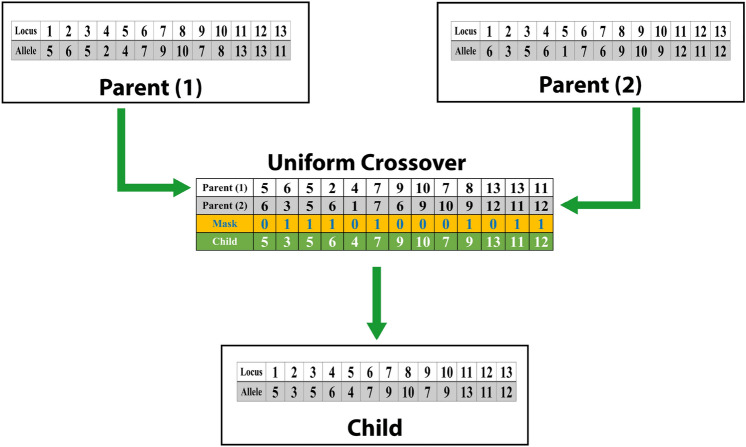
Two parent individuals, each characterized by their respective genotypes, combine their genetic information through a uniform crossover process to produce a child individual.

The proposed complex detection algorithm incorporates an evolutionary approach, utilizing a recombination operator referred to as $r_{pc}:I_1\times I_2\rightarrow I$. This operator, known as canonical uniform crossover $r_{pc}$, merges topological information from two parent individuals, denoted as $I_1$ and $I_2$, to generate an offspring individual. Figure 5 visually illustrates this process by showcasing the graph structures and genotypes of the two parent individuals, highlighting how genetic information from both parents is combined to create a child individual. This method enables the transfer of desirable traits and characteristics from the parent individuals to the newly generated offspring. Mathematically, the topological-based uniform crossover is formalized as follows:

For each gene *j* in the chromosome of individual *i*, where $i\in \{1,2,\dots ,\mu \}$ and $j\in \{1,2,\dots ,n\}$:

21 $$\begin{aligned} \begin{aligned}&I_{i,j}={\left\{ \begin{array}{ll} I_{1,j} & \text {if } \chi _j \le 0.5\\ I_{2,j} & \text {otherwise}\\ \end{array}\right. } \end{aligned} \end{aligned}$$

In this equation, $I_{i,j}$ represents the gene at position *j* in the chromosome of individual *i*. The choice of which parent’s gene to inherit depends on a random variable $\chi _j$, where if $\chi _j$ is less than or equal to 0.5, the gene from $I_1$ is selected, and if $\chi _j$ is greater than 0.5, the gene from $I_2$ is chosen. This uniform crossover mechanism ensures a balanced combination of genetic material from both parents during the creation of offspring, promoting the inheritance of favorable characteristics in the evolutionary process.

### The proposed GO-based heuristic mutation: enhancing genetic diversity through GO integration

In this paper, we present the Functional Similarity-Based Protein Translocation Operator (FS-PTO), a novel heuristic method designed to enhance the identification of functional protein complexes within PPI networks. The FS-PTO improves detection accuracy by strategically evaluating and reallocating proteins based on their functional roles and connectivity. This operator uses functional similarity, denoted as $FS_{BMA}$, to guide the reassignment of proteins between complexes, particularly targeting those proteins that exhibit low functional similarity, known as weak proteins. Such proteins can undermine the functional coherence of their current complexes due to their mismatched functional attributes. The FS-PTO refines the network by reassigning weak proteins to complexes where their functional profiles align more closely with the other proteins. By integrating these weak proteins into more suitable complexes, the functional efficiency of the receiving complexes is enhanced. This realignment promotes synergistic interactions among proteins with similar functions, thereby optimizing the overall performance and stability of the complexes.

To understand how the FS-PTO operates, consider a set of proteins, $I = \{I_1, I_2, \dots , I_N\}$, and a complex structure $\mathcal {C}$ consisting of *K* complexes, $\{C_1, C_2, \dots , C_K\}$. Each protein, $P_i$, is initially evaluated based on its functional similarity to the complex *C* to which it is currently assigned.

The functional similarity, denoted as $FS_{BMA}$, between a protein $P_j$ and a complex *C* is determined by aggregating the similarity scores between $P_j$ and each member protein $P_{k^\prime }$ within the complex *C*. This is expressed as:

22 $$\begin{aligned} \text {F\_Intra}(P_j, C) = \sum _{P_{k'} \in C} FS_{BMA}(P_j, P_{k'}) \end{aligned}$$

This measure indicates how well protein $P_j$ integrates with the existing members of its current complex. On the other hand, the inter-complex functional similarity $FS_{BMA}$ is calculated for each other complex $C_j$ (where $C_j \ne C_i$) as:

23 $$\begin{aligned} \text {F\_Inter}(P_j, C_j) = \sum _{P_{k^\prime } \in C_j} FS_{BMA}(P_j, P_{k^\prime }) \end{aligned}$$

This calculation helps in pinpointing the complex $C_j$ where $P_j$ might be reassigned to achieve better functional alignment.

The decision to reassign a protein is governed by the following criteria:

24 $$\begin{aligned} I_{i,j} = {\left\{ \begin{array}{ll} \arg \max _{C \in \mathcal {C}} \left\{ \sum _{P_{k^\prime } \in C} FS_{BMA}(P_j, P_{k^\prime }) \right\} & \text {if } r \le p_m \\ I_{i,j} & \text {otherwise} \end{array}\right. } \end{aligned}$$

This equation aims to find the complex *C* that maximizes the aggregate functional similarity score for protein $P_j$. The reallocation decision also considers a random value *r* compared to a mutation probability $p_m$, influencing whether a protein should be reassigned to a new complex to enhance functional homogeneity. An outline of the proposed FS-PTO heuristic mutation operator is presented in algorithm 1.

**Algorithm 1:**
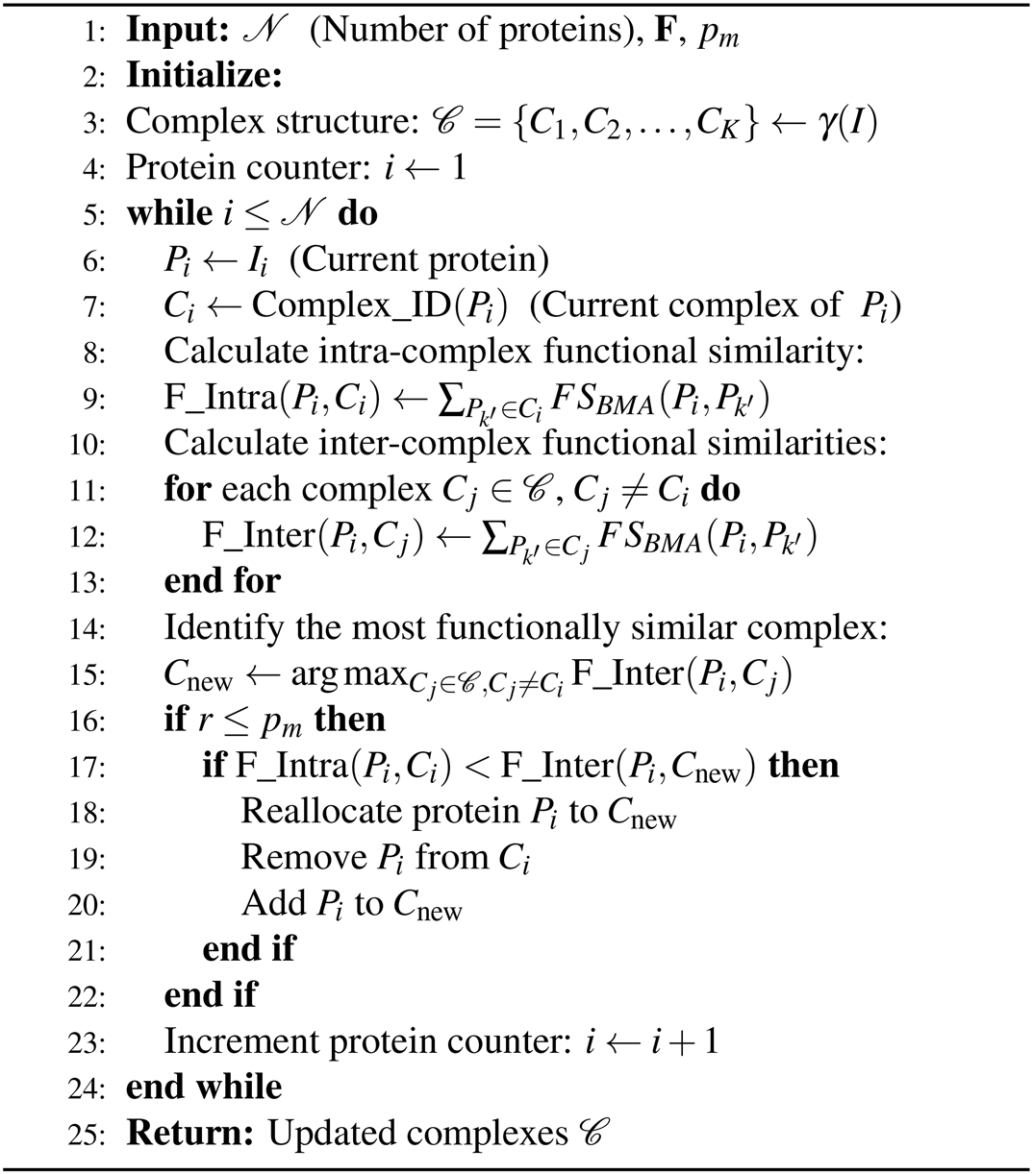
Functional similarity-based protein translocation operator (FS-PTO).

The FS-PTO algorithm is designed to optimize the assignment of proteins to complexes based on their functional similarities. The algorithm works by iterating through each protein and assessing both its current intra-complex and inter-complex functional similarities. Intra-complex functional similarity is calculated for each protein within its current complex. This value represents the functional similarity between the protein and the other proteins in the same complex. On the other hand, inter-complex functional similarity is calculated for each protein with respect to all other complexes. This value helps identify the complex that has the highest functional similarity to the protein.

For each protein, the algorithm compares the intra-complex functional similarity with the inter-complex functional similarity. If the protein’s intra-complex similarity is lower than its inter-complex similarity, the protein is reassigned to the complex with the highest inter-complex similarity. The reassignment involves removing the protein from its current complex and adding it to the new complex. The process continues for each protein until all proteins have been evaluated, resulting in updated complexes.

### Comparative analysis of topological and biological information in protein complex detection

**Fig. 6:**
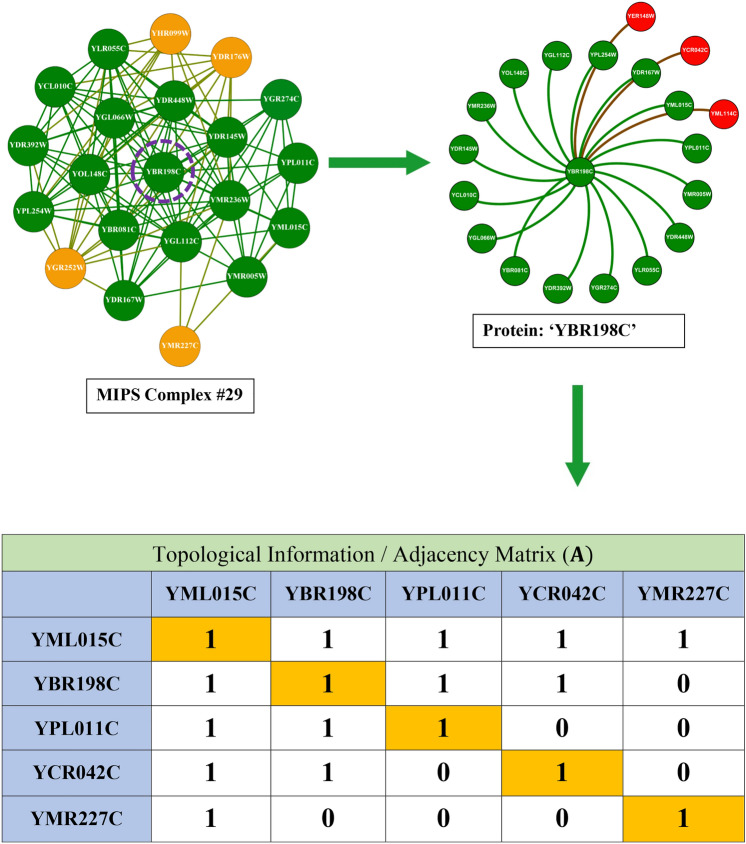
This illustration depicts the interactions of protein ’YBR198C’ within a 20-protein MIPS benchmark complex. Proteins highlighted in green directly interact with ’YBR198C’ by intra-connections, whereas proteins highlighted in yellow do not. Proteins highlighted in red belong to different complexes but are connected to ’YBR198C’ by inter-connections. The bottom section presents the adjacency matrix for the green (’YML015C’, ’YPL011C’), red (’YCR042C’), and yellow (’YMR227C’) proteins.

The corpus of existing literature predominantly anchors its methodology on the utilisation of topological data for the identification of protein complexes. This topological information fundamentally concerns the structural dynamics and connectivity patterns inherent within protein networks. To elucidate this concept, we refer to Fig. 6 in our study, where we selected complex #29 from the MIPS database, comprising 20 distinct proteins. The protein YBR198C emerges as a focal point due to its extensive connectivity within the complex.

**Fig. 7:**
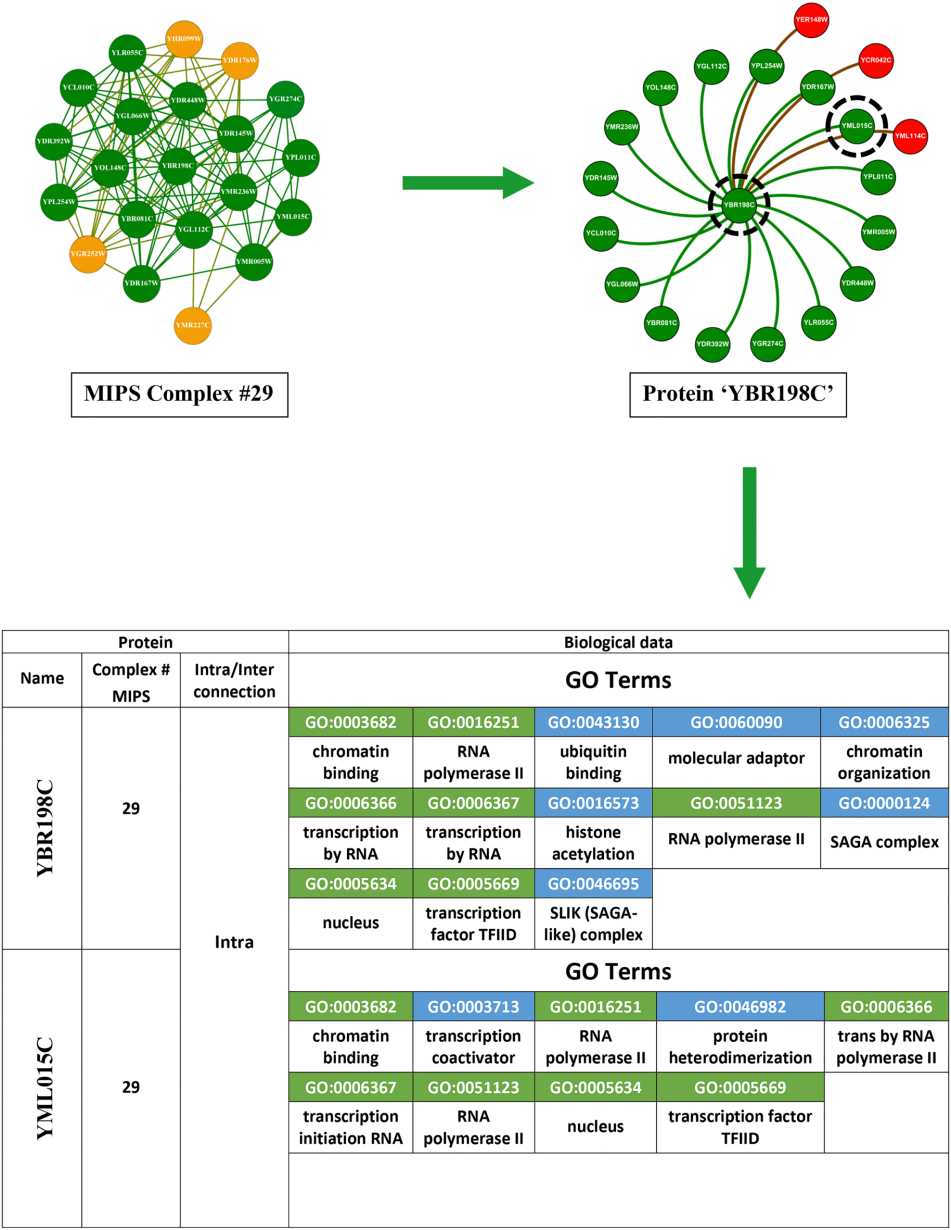
GO terms associated with both proteins YBR198C and YML015C. Shared GO terms are highlighted in green, while distinct GO terms for each protein are highlighted in blue.

**Fig. 8:**
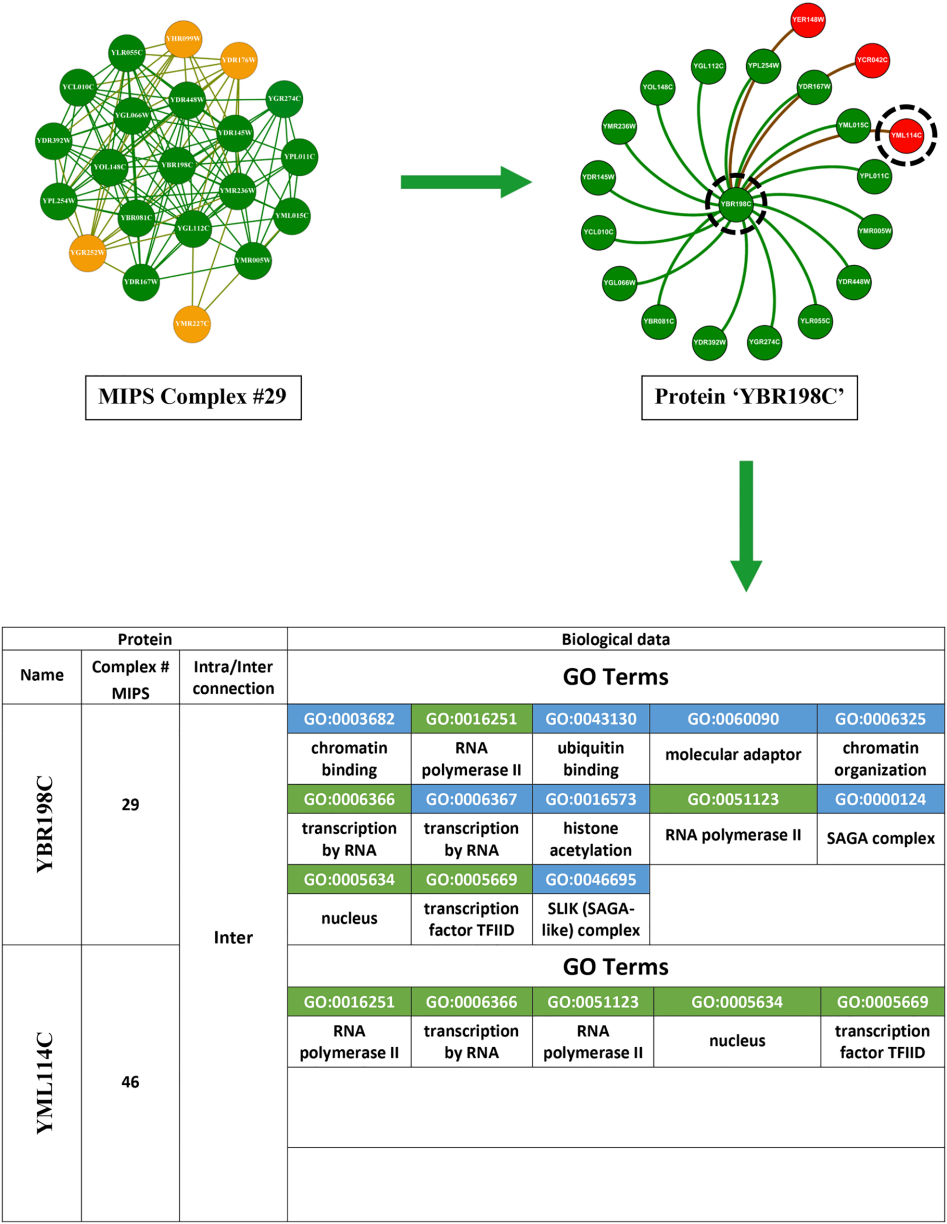
GO terms associated with both proteins YBR198C and YML114C. Shared GO terms are highlighted in green, while distinct GO terms for each protein are highlighted in blue.

**Fig. 9:**
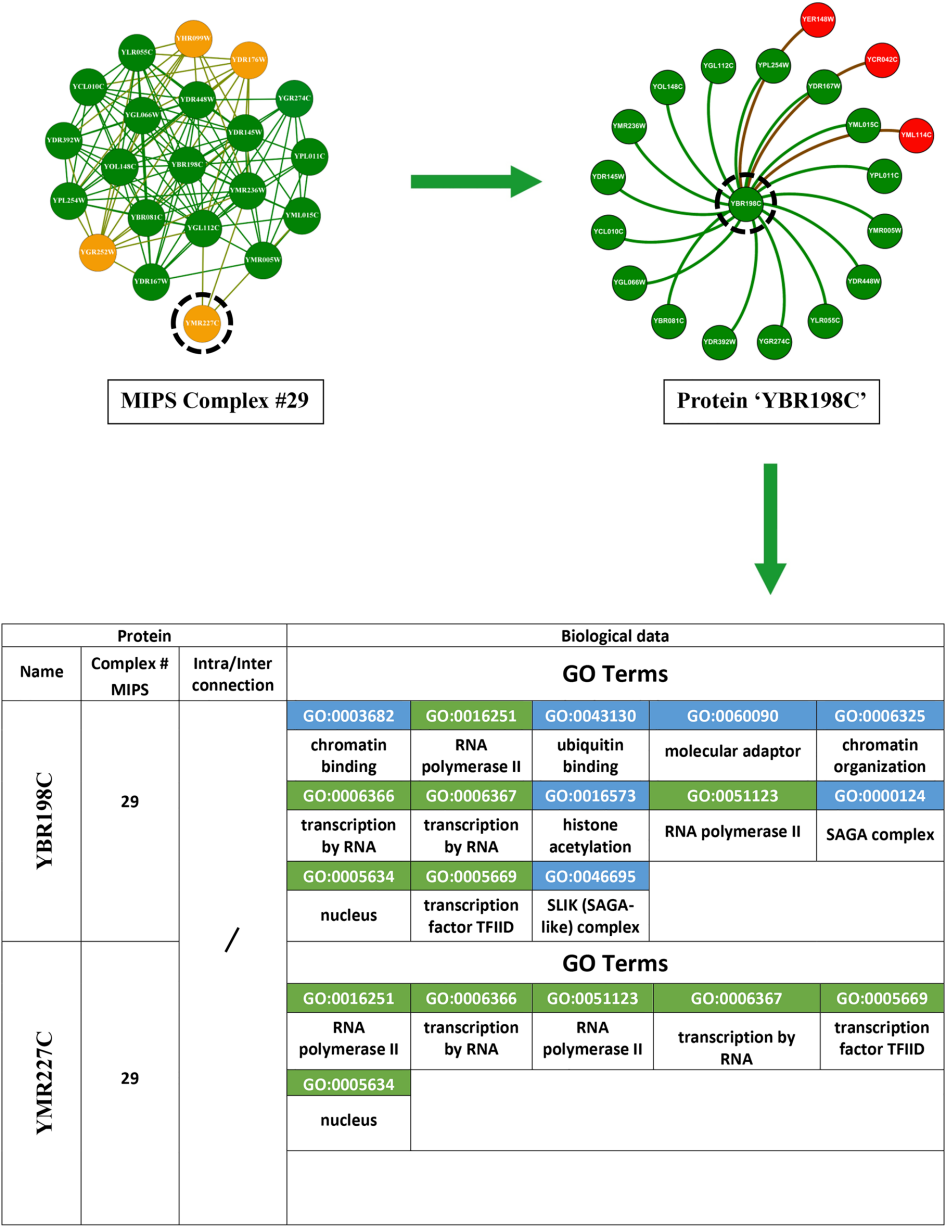
GO terms associated with both proteins YBR198C and YMR227C. Shared GO terms are highlighted in green, while distinct GO terms for each protein are highlighted in blue.

Protein YBR198C is centrally involved, displaying internal linkages with 16 other proteins and engaging in external connections with three additional proteins from a disparate complex. Within our visual representation, proteins affiliated with the same complex are marked in green, whereas those associated with different complexes are highlighted in red. For a more granular analysis, consider three illustrative cases involving YBR198C: Firstly, the protein YML015C, which resides within the same complex, is connected to YBR198C, as evidenced by a ’1’ in the corresponding cell of the adjacency matrix in the lower section of Figure 6, indicating the presence of a linkage. Conversely, a ’0’ denotes the absence of such a connection. Secondly, another protein, external to the complex yet connected to YBR198C, similarly exhibits a connection value of ’1’. Thirdly, YMR227C, also within the same complex but not linked to YBR198C, is represented in yellow, with the adjacency matrix showing a connection value of ’0’. Existing methodologies that depend solely on topological information encounter limitations, exemplified by their inability to associate YMR227C with YBR198C due to the lack of direct connectivity. This highlights a significant shortfall in the capability of these topological approaches to discern protein complexes with precision. In contrast, our investigation pivots towards leveraging biological information to unearth protein complexes. Specifically, we have analyzed the interactions between YBR198C and YML015C using the SGD to extract their GO terms. The results, depicted in Fig. 7, reveal that these proteins share seven GO terms, represented in green, affirming their functional congruence. Another instance involves YML114C, associated with a different complex but sharing five GO terms with YBR198C, as illustrated in Fig. 8. Additionally, YMR227C, while part of the same complex and unlinked to YBR198C, shares six GO terms, as demonstrated in Fig. 9. These instances significantly validate the efficacy of our proposed method, which discerns protein complexes not merely through structural data but through a profound understanding of biological interactions and functional similarities. Despite the inherent noise and variability in protein network data, our approach offers a robust framework for accurately identifying protein complexes, underlining the critical role of biological data in enhancing the precision of complex detection in PPI networks.

**Fig. 10:**
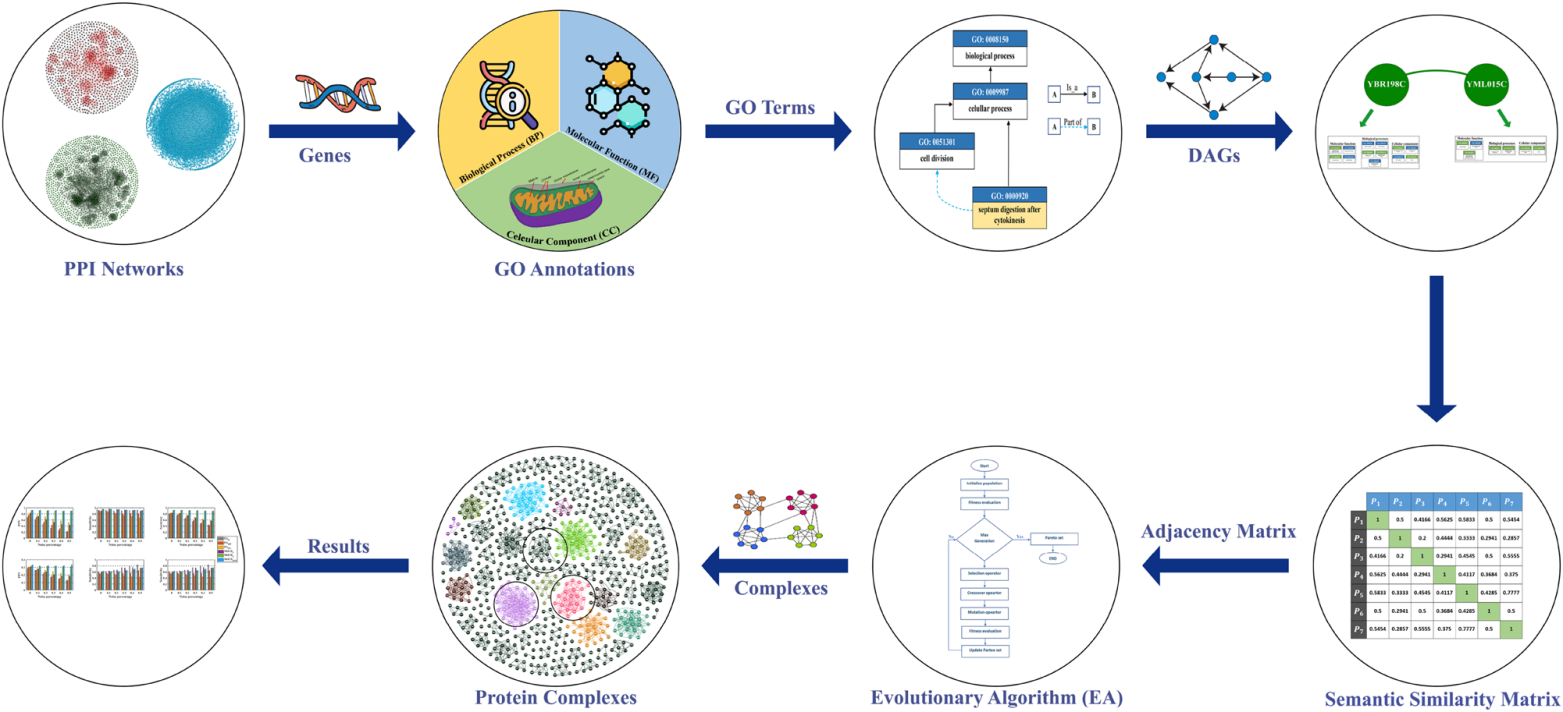
The methodology framework comprises a sequence of steps aimed at assessing gene similarity. Initially, we obtain GO annotations via the SGD. Subsequently, we generate a DAG for each GO term sourced from the GO. We then calculate gene similarity and incorporate the resultant similarity matrix into our method as vital elements of an evolutionary-based algorithm meticulously crafted for detecting complex structures. Lastly, we conduct an evaluation to gauge the quality of the detected complexes.

The methodology presented in this paper, illustrated in Fig. 10, outlines a structured and comprehensive framework for systematically evaluating the similarity between GO terms. The framework consists of several key stages, each contributing to the primary objective of accurately detecting and evaluating biological complexes. The process begins with obtaining GO annotations from the SGD for a given yeast dataset. These annotations provide essential information about the functional aspects of genes, categorizing them into three primary ontologies: BP, MF, and CC. This initial step establishes a foundation for understanding the functional roles of genes and their relationships within various biological processes.

Once the GO annotations are acquired, the next step involves constructing a DAG for each GO term. The DAG captures the hierarchical relationships between different GO terms, showing how they are functionally dependent on one another. This graph structure is critical for visualizing the complexity of gene functions and their interconnections, offering insight into how various biological processes or functions are related.

With the GO annotations and DAG in place, the methodology proceeds to the calculation of gene similarity based on these annotations. This step evaluates the functional similarity between gene pairs, producing a similarity matrix that quantifies their degree of similarity. This matrix plays a central role in the framework, as it serves as the key input for the subsequent stage of the process.

The similarity matrix is then incorporated into our model, which is specifically designed to detect protein complexes within biological data. EAs, inspired by natural selection, are employed to iteratively refine solutions, identifying protein complexes or gene groups that share functional similarities. By integrating the gene similarity matrix, the algorithm improves its ability to detect complex structures with more accuracy.

Finally, the output generated by our model undergoes a rigorous evaluation phase. During this phase, the identified complexes are assessed using various metrics, such as accuracy and biological relevance.

## Experiments and evaluation

In this section, we systematically assess the quality of the complexes generated by our proposed approach through a three-phase evaluation process. First, we compare the performance of our model against existing heuristic state-of-the-art complex detection methods to establish its baseline effectiveness. Next, we evaluate the proposed model’s effectiveness by benchmarking it against heuristic-based EA models, providing insights into its relative performance within the EA domain. In the final phase, we evaluate the robustness of our approach by introducing or removing interactions, and comparing the results with other EA-based models to demonstrate the stability and reliability of our method under varying network conditions.

### Datasets

To conduct a rigorous performance evaluation, we utilized two PPI networks meticulously derived from the yeast species Saccharomyces cerevisiae. The first PPI network dataset, known as Yeast-D1, underwent meticulous curation led by Gavin et al.^48^. This process involved the careful selection and validation of PPIs. Subsequently, the curated dataset underwent further refinement through a rigorous filtration process, guided by Zaki et al.^49^. The outcome is a highly reliable and accurate network comprising a notable $m=4687$ interactions, involving a total of $n=990$ distinct proteins.

**Fig. 11:**
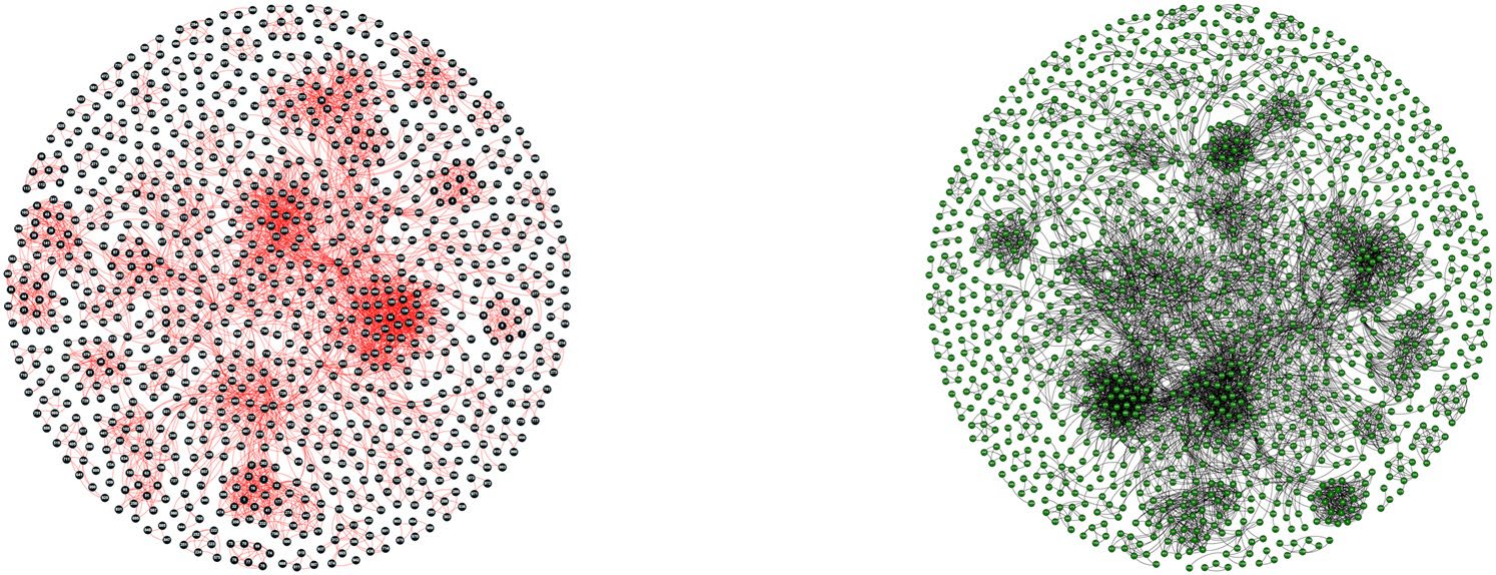
Two distinct PPI networks: Yeast-D1 (on the left) and Yeast-D2 (on the right). These networks represent intricate biological interactions among proteins, providing valuable insights into cellular processes and functions.

One noteworthy aspect of the Yeast-D1 dataset is the variability in the number of interactions per protein, denoted as $m_i$. This parameter displays significant diversity, ranging from a minimum of 1 to a maximum of 52. This variation offers valuable insights into the connectivity and centrality of different proteins within the yeast protein interaction network. Simultaneously with the curation and refinement of Yeast-D1, a comprehensive annotation process unfolded for the 990 proteins. This meticulous annotation involved systematically assigning GO terms to each protein, facilitating a deeper understanding of their functional roles. Specifically, these proteins were meticulously annotated with 5645 BP terms, 4904 CC terms, and 3434 MF terms. These GO annotations provide a rich resource for characterizing the functional attributes of proteins within the Yeast-D1 dataset. Transitioning to the second PPI network dataset, referred to as Yeast-D2, it represents a comprehensive amalgamation of yeast protein interactions derived from six distinct experimental sources. This composite dataset was intentionally compiled to encompass a broad spectrum of interactions within the yeast species, aiming to provide a more holistic view of the yeast protein interaction network. Following the compilation of Yeast-D2, an additional filtration step, guided by Zaki et al.^49^, was applied to enhance data quality. This filtration resulted in a refined subset comprising 1443 proteins with a notable count of 6993 interactions. Similar to Yeast-D1, the number of interactions per protein, denoted as $m_i$, exhibits a considerable range, spanning from a minimum of 1 to a maximum of 59. This variability in interaction counts is indicative of the diversity and complexity of protein interactions within Yeast-D2. Figure 11 illustrates the Yeast-D1 and Yeast-D2 PPI networks, which were visualized using Gephi^50^, an open-source network visualization tool. The construction process involved importing the PPI dataset as an edge list into Gephi, where each edge represents an interaction between two proteins. To enhance interpretability, a Fruchterman-Reingold layout was applied, which simulates a physical system where nodes repel each other while edges act as springs pulling connected nodes together. This results in a visually balanced distribution of nodes that highlights interaction patterns effectively. Specifically, each node represents a protein, while edges represent PPIs. For example, if proteins P1, P2, and P3 interact such that P1 interacts with both P2 and P3, the resulting network would display P1 positioned in a way that maintains an optimal balance between repulsive and attractive forces, ensuring clarity in structural representation. The final visualization in Fig. 11 effectively captures the structural properties and interaction densities of both Yeast-D1 and Yeast-D2.

Concurrent with the refinement of Yeast-D2, an extensive annotation effort was undertaken to associate functional attributes with the 1443 proteins. This annotation process involved systematically assigning GO terms to each protein, resulting in a comprehensive repertoire of functional annotations. Specifically, these proteins were meticulously annotated with 8111 BP terms, 6846 CC terms, and 4904 MF terms. These annotations provide valuable insights into the functional roles and cellular locations of proteins within the Yeast-D2 PPI network, making it a valuable resource for studying yeast biology and protein interactions in detail. To assess the effectiveness and reliability of our proposed model in accurately predicting protein complexes, we performed a comprehensive validation using two meticulously curated benchmark datasets: Complex-D1 and Complex-D2. Both datasets were sourced from the well-regarded MIPS catalog ^38^. Complex-D1, the first benchmark dataset, consists of 859 proteins organized into 81 distinct complexes. These complexes vary in size from 6 to 38 proteins, with an average of approximately 8.9 proteins per complex. Notably, this dataset includes Yeast-D1, which contains a comprehensive collection of 701 known proteins. In contrast, Complex-D2 is a more exclusive dataset, featuring 162 carefully selected complexes that range from 4 to 266 proteins, totaling 3125 proteins. Within Complex-D2, Yeast-D2 accounts for 680 proteins. The primary distinction between Complex-D1 and Complex-D2 lies in their structural attributes. The complexes in Complex-D1 are inherently disjoint, meaning there is no overlap between any pair of complexes (i.e., $C_i^*\cap C_j^*= \varnothing$). Conversely, Complex-D2 presents a higher level of complexity, with 190 complexes exhibiting overlapping features. This overlap is due to 1255 proteins that are shared among multiple complexes, resulting in instances where ($C_i^*\cap C_j^*\ne \varnothing$) for many of the complexes.

### Evaluation measures

In the context of biological networks where standard or reference complexes are established, evaluating the quality of identified protein complexes is crucial. This evaluation is commonly performed using well-known statistical measures such as *recall*, *precision*, and $\text {F-score}$, as described by Tan et al.^51^. These metrics provide a systematic way to compare the detected protein complexes with the established standard complexes, thereby offering insights into the accuracy and relevance of the findings.

To elaborate, for each protein complex identified in the PPI network, denoted as $C_j$, the comparison with a corresponding standard complex, denoted as $C_i^*$, involves the calculation of several key sets. The True Positives (*TP*) represent the set of proteins that are correctly identified, meaning they are present in both the detected complex $C_j$ and the standard complex $C_i^*$. This set indicates the degree to which the discovered complex overlaps with the known standard.

In contrast, False Positives (*FP*) refer to the set of proteins that are included in the detected complex $C_j$ but are not part of the standard complex $C_i^*$. This set reflects the extent of incorrect or extraneous proteins that have been erroneously grouped into the complex during the detection process. On the other hand, False Negatives (*FN*) represent the proteins that are part of the standard complex $C_i^*$ but have been missed or excluded from the detected complex $C_j$. This measure is crucial for understanding the completeness of the detected complex in capturing the entirety of the standard complex.

25 $$\begin{aligned} Recall= & \frac{TP}{TP + FN} \end{aligned}$$

26 $$\begin{aligned} Precision= & \frac{TP}{TP + FP} \end{aligned}$$

27 $$\begin{aligned} {F\text {-}}score= & 2 \times \frac{Recall \times Precision}{Recall + Precision} \end{aligned}$$

The evaluation of the proposed model involves considering the Jaccard similarity score, represented by Eq. (28), as a means to assess the similarity between a predicted complex $C_j$ and a benchmark complex $C_i^*$. This score is computed as the ratio of the number of proteins that are shared by both $C_i^*$ and $C_j$ to the total number of proteins in the set that contains all proteins from $C_i^*$ and $C_j$. By utilizing this Jaccard similarity score, the degree of overlap and similarity between the predicted and benchmark complexes can be quantitatively measured, providing valuable insights into the performance of the proposed model.

28 $$\begin{aligned} J(C_i^*,C_j)=\frac{\vert C_i^*\bigcap C_j\vert }{\vert C_i^*\bigcup C_j\vert } \end{aligned}$$

In the context of our study, we employ a crucial metric known as the Jaccard similarity coefficient to assess the similarity between a benchmark complex, denoted as $C_i^*$, and a predicted complex, denoted as $C_j$. This metric, represented as $J(C_i^*,C_j)$, serves as a fundamental measure of agreement between the two complexes. The core of our evaluation lies in the application of a specific criterion: if the Jaccard similarity coefficient $J(C_i^*,C_j)$ surpasses or equals a predetermined threshold known as $\delta$, then we consider the predicted complex $C_j$ as a valid prediction for the benchmark complex $C_i^*$. In essence, the value of $\delta$ serves as a quantitative indicator, delineating the level of concordance required between a predicted complex and a complex drawn from our benchmark dataset.

### Algorithm parameter settings

The algorithms proposed in this study, along with all EA-based approaches analyzed herein, have been configured according to the standard parameters outlined in Table  1.

**Table 1:** Experimental settings and parameter values for EAs.

Parameter	Description/value
Population size ($\mu$)	The size of the population is set to 100, following the recommended practice of ensuring a sufficiently large and diverse population for effective evolutionary search. A larger population helps explore the search space comprehensively.
Maximum number of generations	The maximum number of generations is predetermined as 100, equivalent to a total of 10,000 function evaluations. This setting controls the termination condition of the evolutionary process, ensuring a finite and bounded search.
Uniform crossover probability (*pc*)	The probability of applying uniform crossover is fixed at a value of 0.8. This reflects a preference for a higher likelihood of generating offspring with well-balanced genetic information inherited from both parents, promoting exploration and exploitation in the search space.
Mutation probability ($p_m$)	The mutation operator, responsible for introducing diversity and facilitating exploration of unexplored regions in the search space, is assigned a probability represented by $p_m$, specifically set to 0.2. This setting controls the likelihood of mutation occurring in each generation.
Proposed heuristic GO-based ($p_m$)	A proposed mutation operator based on Gene Ontology (GO) is incorporated into the algorithms, also assigned a probability of $p_m=0.2$. This specialized mutation operator aims to inject domain-specific knowledge into the search process.
Evaluation metrics	The evaluation metrics discussed in Section “Evaluation measures” are rigorously analyzed and reported based on the average results obtained from conducting 30 independent runs. This approach of averaging outcomes across multiple runs provides a comprehensive and robust assessment of the algorithms’ performance, ensuring that the reported results are statistically significant and representative of their overall effectiveness in finding optimal solutions.

### Assessing the robustness of the proposed GO-based MOEA against Noisy PPI Networks

The reliability of a PPI network is a critical concern in the field of systems biology, primarily due to the substantial noise present in high-throughput experiments. High-throughput experiments are known to introduce a high rate of false positives, which can result in spurious inter-complex interactions within the network. Conversely, there is also the issue of missing genuine protein interactions that should be present in a reliable PPI network. Researchers such as^26,31^ have made significant contributions in addressing these challenges by developing algorithms aimed at assessing the consistency and effectiveness of algorithms designed to detect protein complexes in PPI networks, even in the presence of noise.

In the studies conducted by^26,31^, the addition and deletion of interactions within PPI networks were performed in a random manner. This approach allowed them to evaluate the robustness and performance of their algorithms under conditions that simulate the inherent noise found in experimental PPI datasets. The insights gained from these investigations have been invaluable in advancing our understanding of how well these algorithms can adapt to real-world scenarios characterized by noise and uncertainties.

In this study, we rigorously evaluate several EAs, including $EA-CS$^31^, $EA-{CS_{mu}}$^37^, *MOEA*/*D*^35^, $MOEA/D_{mu}$^37^, and our model $MOEA-GO_{FS-PTO}$, by testing them on synthetic PPI networks. To simulate real-world conditions, we introduce varying levels of noise into the Yeast-D1 and Yeast-D2 networks by adding or removing interactions between proteins. Specifically, we adjust the proportion of interactions altered to $10\%, 20\%, 30\%, 40\%, and 50\%$. For each proportion, we create 10 distinct synthetic networks. The interactions in these networks are modified by either adding or removing them from proteins chosen based on different criteria: randomly selected proteins, targeting those with the most interactions, or focusing on those with the fewest interactions.

To quantitatively evaluate the impact of these noise types on the PPI networks, we collected statistics and reported them in four separate tables: Tables 2, 3, 4 and 5 summarize the impact of interaction modifications. Tables 2 and 3 cover the addition of spurious interactions, while Tables 4 and 5 addresses the removal of true interactions. In all tables, *m* denotes the total interactions, ($\vert n \vert _{d=1}$) shows proteins with only one interaction, and ($d_{Avg}$) represents the average number of interactions per protein.

**Table 2:** Statistics on the impact of adding spurious interactions to the Yeast-D1 dataset.

Noise	$Add_{Random}$			$Add_{HighDegree}$			$Add_{LowDegree}$		
	*m*	$\vert n \vert _{d=1}$	$d_{Avg}$	*m*	$\vert n \vert _{d=1}$	$d_{Avg}$	*m*	$\vert n \vert _{d=1}$	$d_{Avg}$
0%	4687	28	9.4687	4687	28	9.4687	4687	28	9.4687
10%	5189	28	10.4828	5065	21	10.2323	4778	20	9.6525
20%	5689	28	11.4929	5443	15	10.9959	4868	10	9.8343
30%	6179	28	12.4828	5821	13	11.7595	4959	7	10.0181
40%	6684	28	13.5030	6199	5	12.5232	5049	6	10.2
50%	7147	28	14.4383	6578	5	13.2888	5140	4	10.3838

**Table 3:** Statistics on the impact of adding spurious interactions to the Yeast-D2 dataset.

Noise	$Add_{Random}$			$Add_{HighDegree}$			$Add_{LowDegree}$		
	*m*	$\vert n \vert _{d=1}$	$d_{Avg}$	*m*	$\vert n \vert _{d=1}$	$d_{Avg}$	*m*	$\vert n \vert _{d=1}$	$d_{Avg}$
0%	6993	92	9.6923	6993	92	9.6923	6993	92	9.6923
10%	7835	92	10.8593	7572	92	10.4948	7113	64	9.8586
20%	8607	92	11.9293	8150	92	11.2959	7234	42	10.0263
30%	9417	92	13.0520	8729	92	12.0984	7354	39	10.1927
40%	10249	92	14.2051	9308	92	12.9009	7474	27	10.3590
50%	11043	92	15.3056	9886	92	13.7020	7595	20	10.5267

**Table 4:** Statistics on the impact of removing true interactions from the Yeast-D1 Dataset.

Noise	$Del_{Random}$			$Del_{HighDegree}$			$Del_{LowDegree}$		
	*m*	$\vert n \vert _{d=1}$	$d_{Avg}$	*m*	$\vert n \vert _{d=1}$	$d_{Avg}$	*m*	$\vert n \vert _{d=1}$	$d_{Avg}$
0%	4687	28	9.4687	4687	28	9.4687	4687	28	9.4687
10%	4249	53	8.5838	4309	28	8.7050	4596	32	9.2848
20%	3851	70	7.7797	3931	28	7.9414	4506	36	9.1030
30%	3480	89	7.0303	3553	29	7.1777	4415	43	8.9191
40%	3191	119	6.4464	3175	30	6.4141	4325	45	8.7373
50%	2899	168	5.8565	2796	35	5.6484	4234	48	8.5535

**Table 5:** Statistics on the impact of removing true interactions from the Yeast-D2 Dataset.

Noise	$Del_{Random}$			$Del_{HighDegree}$			$Del_{LowDegree}$		
	*m*	$\vert n \vert _{d=1}$	$d_{Avg}$	*m*	$\vert n \vert _{d=1}$	$d_{Avg}$	*m*	$\vert n \vert _{d=1}$	$d_{Avg}$
0%	6993	92	9.6923	6993	92	9.6923	6993	92	9.6923
10%	6332	127	8.7762	6414	92	8.8898	6873	95	9.5260
20%	5749	173	7.9681	5836	92	8.0887	6752	101	9.3583
30%	5235	178	7.2557	5257	92	7.2862	6632	107	9.1920
40%	4721	236	6.5433	4678	93	6.4837	6512	119	9.0256
50%	4308	241	5.9709	4100	100	5.6826	6391	131	8.8579

### Complex detection performance: GO-based multi-objective evolutionary algorithm against state-of-the-art methods

This section presents a comprehensive performance comparison between the proposed GO-based MOEA and state-of-the-art heuristic and evolutionary-based methods for complex detection. The experimental evaluation begins with the reporting of results obtained from the proposed GO-based MOEA, which are compared to established heuristic methods, including MCODE^27^, OCG^52^, LC^53^, NDOCD^54^, RNSC^55^, ELC^56^, CPM^57^, and MCL^58^ as documented in Table  6. The evaluation focuses on the Yeast-D1 and Yeast-D2 datasets. In this analysis, the GO-based mutation operator assumes the role of a ’background heuristic’ operator, with a low probability of occurrence set at $p_m=0.2$. Additionally, to establish a successful match between a predicted complex and a true complex, a minimum overlap of at least $20\%$ is required between their respective constituent elements, denoted as the overlapping score $\delta =0.2$.

**Table 6:** Performance comparison at the complex level: Evaluating *Recall*, *Precision*, and *F*-score with a $\delta =0.2$ threshold. The comparison encompasses established heuristic-based complex detection algorithms alongside the proposed GO-based MOEA. The proposed heuristic mutation operator is set to a low probability of occurrence, $p_m=0.2$. Outstanding results are marked in bold.

Algorithm	Yeast-D1			Yeast-D2		
	Recall	Precision	F-score	Recall	Precision	F-score
MCODE	0.6700	0.6250	0.6467	0.3410	0.3650	0.3526
OCG	0.8380	0.6150	0.7094	0.6000	0.3450	0.4381
LC	0.4950	0.0410	0.0757	0.6995	0.0800	0.1436
NDOCD	0.7830	0.7000	0.7392	0.4225	0.4190	0.4207
RNSC	0.8490	0.2650	0.4039	0.4850	0.1560	0.2361
ELC	0.5910	0.6479	0.6181	0.2855	0.3890	0.3293
CPM	0.5850	0.6170	0.6006	0.3050	0.3955	0.3444
MCL	0.8230	0.5390	0.6514	0.1900	0.2920	0.2302
$\text {MOEA}\text{- }GO_{FS-PTO}$	**0.9436**	**0.7593**	**0.8209**	**0.8953**	**0.5702**	**0.6839**

**Fig 12:**
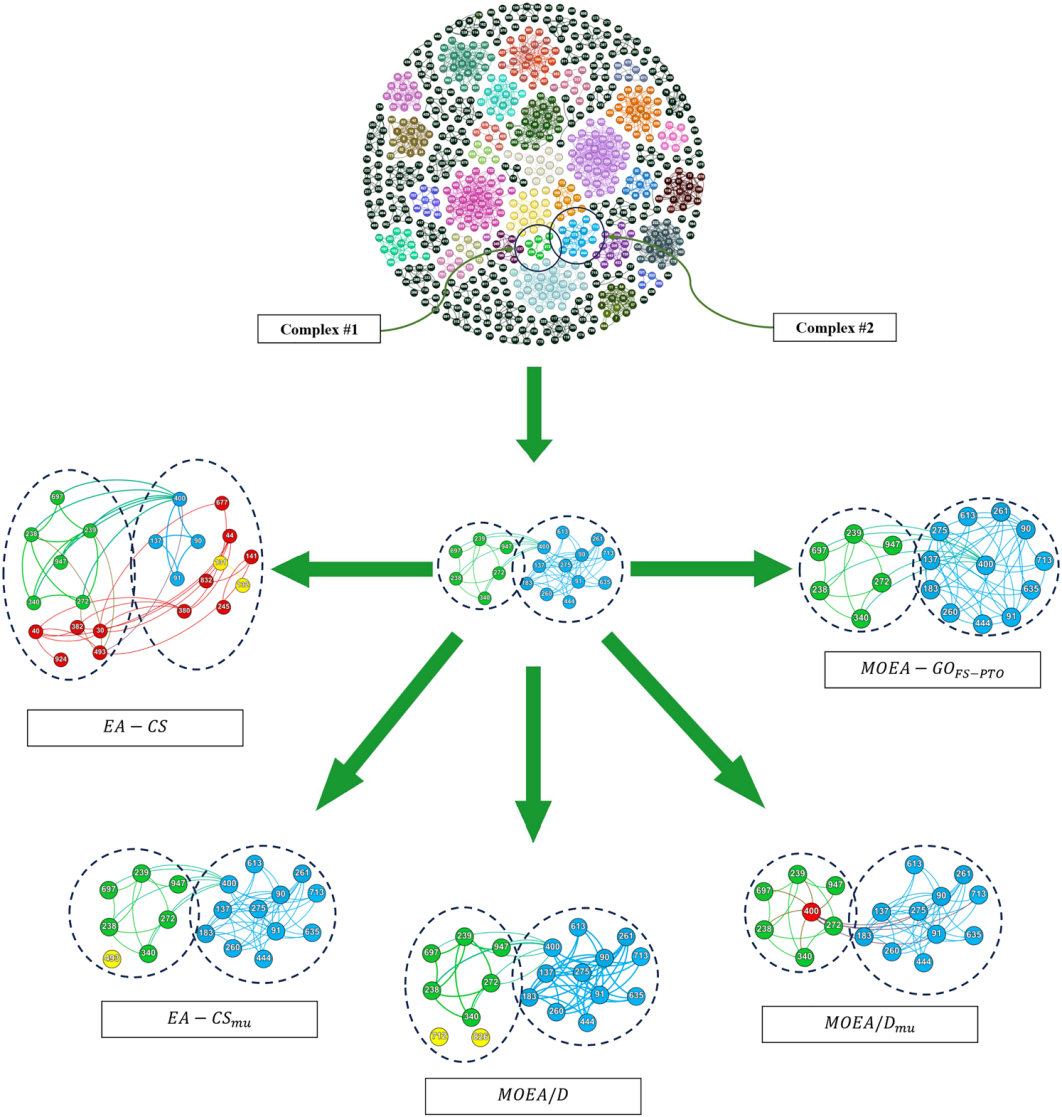
Illustration depicting the evaluation process of protein complex detection methods using representative complexes sourced from the MIPS database. Color annotations show detection accuracy: green/blue indicating correct detection, red for erroneous inclusions, and yellow for arbitrary placements.

Moreover, we present comprehensive results in Tables  7 and  8, which demonstrate the performance of the latest advancements in single EAs introduced by^31^ and^37^, respectively. Tables  7 and  8 specifically focus on comparing the performance of these advanced EAs against the canonical EAs with single-objective models proposed by^31^, as well as their corresponding heuristic-based EAs introduced in^37^. The evaluated models include conductance (CO), community score (CS), expansion (EX), internal density (ID), normalized cut (NC), and ratio cut (RC). Through these comparisons, we aim to assess the efficacy of the proposed enhancements. Building on these findings, the culmination of our investigation, depicted in Table  9, serves as a comprehensive portrayal of the advancements achieved through the utilization of sophisticated multi-objective EAs. These algorithms, notably through the incorporation of a heuristic-based mutation operator proposed in^37^. This augmentation represents a significant stride towards enhancing the performance and efficacy of MOEAs in tackling complex optimization challenges. Further extending this investigation, our proposed MOEA framework, with the integration of the gene ontology-based mutation operator, the FS-PTO operator, we have successfully identified several instances of protein complexes with distinct biological importance. As depicted in Fig. 12, the protein complexes under examination were sourced from the MIPS database^38^, comprising a total of 81 accurately annotated complexes. Our investigation aimed to assess the efficacy of both the proposed methodology and conventional approaches in identifying these complexes. To elucidate the intricacies of complex structures, two representative complexes from the MIPS database were selected, and their intra- and inter-connectivity were magnified for detailed scrutiny. The methodology proposed by Pizzuti et al.^31^ underwent rigorous evaluation. However, it became evident that this approach exhibited inconsistencies in protein detection. Notably, proteins highlighted in green and blue denote accurate identification in alignment with the MIPS database, whereas those in red signify erroneous inclusions within the complex. Furthermore, proteins shaded in yellow indicate arbitrary placement within the complex, lacking meaningful associations with other constituent proteins. In evaluating the $EA-CS_{mu}$ method, it was observed that while the operator proposed in^37^ contributed to enhancing the algorithm’s proficiency in identifying protein complexes, it also indiscriminately included protein ($\#493$) within the complex. Similarly, the utilization of single-objective algorithms necessitated exploration into multi-objective algorithms such as *MOEA*/*D* as proposed in^35^ for complex detection. However, this approach also yielded false positives, with proteins numbered ($\#712$ and $\#826$) erroneously integrated into the detected complexes. Subsequently, employing the multi-objective algorithm in conjunction with the operator proposed in^37^, denoted as $MOEA/D_{mu}$, demonstrated improved accuracy in protein detection. Nonetheless, an issue arose wherein protein ($\#400$) migrated from the second complex to the first, resulting in misallocation. Finally, the efficacy of the proposed algorithm, $\text {MOEA}\text{- }GO_{FS-PTO}$, was evaluated. Leveraging the robustness of the proposed operator alongside biological insights derived from GO, this methodology successfully identified all correct proteins within both the first and second complexes. This unequivocally underscores the superiority of the proposed approach rooted in GO for protein complex detection, surpassing the performance of traditional methods.

In Table  9, we meticulously juxtapose the results derived from these advanced MOEAs against several benchmarks. Firstly, we compare them against the conventional MOEA/D framework, initially proposed by^59^. This juxtaposition sheds light on the extent of improvement achieved through the integration of the heuristic-based mutation operator. Furthermore, we contrast the performance of these refined MOEAs with other state-of-the-art approaches, namely, the heuristic-based multi-objective MOEAs advocated by^37^. It is imperative to note that the parameter configurations utilized in our study remain consistent across all evaluated methodologies. This alignment ensures a fair and unbiased comparison, facilitating a clearer understanding of the performance disparities observed.

**Table 7:** Performance comparison was conducted at a complex level with an overlapping score threshold of $\delta =0.2$, focusing on key metrics including *Recall*, *Precision*, and *F*-score. The evaluated algorithms consisted of canonical single-objective EAs as proposed in^31^, and the proposed GO-based MOEA. All of these EAs were configured according to the settings used in this study. Outstanding results are marked in bold.

Algorithm	Yeast-D1			Yeast-D2		
	Recall	Precision	F-score	Recall	Precision	F-score
$\text {EA}\text{- }CS$	0.8718	0.7232	0.7902	0.8133	0.4861	0.6082
$\text {EA}\text{- }EX$	0.7910	0.7009	0.7430	0.7740	0.4790	0.5916
$\text {EA}\text{- }RC$	0.7128	0.7280	0.7202	0.7213	0.4948	0.5868
$\text {EA}\text{- }NC$	0.7026	0.7319	0.7166	0.7080	0.5014	0.5870
$\text {EA}\text{- }ID$	0.7269	0.6540	0.6882	0.7053	0.4413	0.5427
$\text {EA}\text{- }Q$	0.7462	0.7006	0.7225	0.7713	0.5003	0.6068
$\text {MOEA}\text{- }GO_{FS-PTO}$	**0.9436**	**0.7593**	**0.8209**	**0.8953**	**0.5702**	**0.6839**

**Table 8:** Performance comparison was conducted at a complex level with an overlapping score threshold of $\delta =0.2$, focusing on key metrics including *Recall*, *Precision*, and *F*-score. The evaluated algorithms consisted of heuristic-based EAs introduced by^37^, and the proposed GO-based EAs. Outstanding results are marked in bold.

Algorithm	Yeast-D1			Yeast-D2		
	Recall	Precision	F-score	Recall	Precision	F-score
$\text {EA}\text{- }CS_{mu}$	0.9000	0.7289	0.8053	0.8360	0.4764	0.6067
$\text {EA}\text{- }EX_{mu}$	0.8321	0.6532	0.7315	0.7773	0.4490	0.5690
$\text {EA}\text{- }RC_{mu}$	0.6244	0.7191	0.6680	0.6807	0.4903	0.5696
$\text {EA}\text{- }NC_{mu}$	0.6577	0.7426	0.6972	0.7107	0.5111	0.5945
$\text {EA}\text{- }ID_{mu}$	0.7474	0.6361	0.6869	0.7140	0.4337	0.5395
$\text {EA}\text{- }Q_{mu}$	0.6615	0.7313	0.6943	0.6813	0.4919	0.5709
$\text {MOEA}\text{- }GO_{FS-PTO}$	**0.9436**	**0.7593**	**0.8209**	**0.8953**	**0.5702**	**0.6839**

**Table 9:** Performance comparison was conducted with an overlapping score threshold of $\delta =0.2$, The compared MOEAs include the canonical MOEA ($\text {MOEA/D}$) from^35^, heuristic-based EAs ($\text {MOEA/D}_{mu}$) from^37^, and the proposed GO-based MOEAs ($\text {MOEA}\text{- }GO_{FS-PTO}$). Outstanding results are marked in bold.

Algorithm	Yeast-D1			Yeast-D2		
	Recall	Precision	F-score	Recall	Precision	F-score
$\text {MOEA/D}$	0.8667	0.7093	0.7628	0.7720	0.4686	0.5749
$\text {MOEA/D}_{mu}$	0.9026	0.6761	0.7579	0.8040	0.4758	0.5825
$\text {MOEA}\text{- }GO_{FS-PTO}$	**0.9436**	**0.7593**	**0.8209**	**0.8953**	**0.5702**	**0.6839**

Tables 10, 11, and 12 provide a detailed overview of our robustness evaluation. This evaluation includes metrics such as *Recall*, *Precision*, and *F*-score. The tables cover the performance across yeast datasets. Additionally, they present the results for the corresponding synthesized noisy networks, allowing for a comprehensive comparison of how well the methods perform under different levels of noise and network perturbations. In contrast, Tables 13, 14, and 15 present results from a different aspect of our robustness assessment, but in this case, we specifically removed true interactions from the networks to evaluate their resilience to such deletions.

**Table 10:** Robustness evaluation in terms of *Recall*, *Precision*, and *F*-score. False interactions are randomly added to protein pairs.

Noise	Algorithm	Yeast-D1			Yeast-D2		
		Recall	Precision	F-score	Recall	Precision	F-score
10%	$EA-CS$	0.4545	0.5196	0.4844	0.3130	0.3201	0.3159
	$EA-CS_{mu}$	0.5462	0.4965	0.5196	0.2769	0.2333	0.2529
	$\text {MOEA/D}$	0.5407	0.5728	0.5362	0.2742	0.2828	0.2690
	$\text {MOEA/D}_{mu}$	0.8231	0.6000	0.6835	0.4227	0.3079	0.3445
	$\text {MOEA}\text{- }GO_{FS-PTO}$	0.9244	0.8766	0.8569	0.8833	0.7009	0.7579
	$EA-CS$	0.3427	0.4534	0.3897	0.2388	0.2889	0.2607
20%	$EA-CS_{mu}$	0.4585	0.4440	0.4508	0.2053	0.1985	0.2014
	$\text {MOEA/D}$	0.4680	0.5652	0.4812	0.2362	0.2814	0.2423
	$\text {MOEA/D}_{mu}$	0.8154	0.6060	0.6870	0.3927	0.2997	0.3284
	$\text {MOEA}\text{- }GO_{FS-PTO}$	0.9231	0.9069	0.8801	0.8713	0.7266	0.7607
	$EA-CS$	0.2713	.04054	0.3242	0.1801	0.2552	0.2102
30%	$EA-CS_{mu}$	0.3726	0.4036	0.3867	0.1724	0.1920	0.1811
	$\text {MOEA/D}$	0.4154	0.5643	0.4348	0.2012	0.2909	0.2161
	$\text {MOEA/D}_{mu}$	0.8128	0.6160	0.6896	0.3867	0.3006	0.3261
	$\text {MOEA}\text{- }GO_{FS-PTO}$	0.9179	0.9096	0.8737	0.8933	0.7178	0.7683
	$EA-CS$	0.1890	0.3217	0.2375	0.1310	0.2134	0.1612
40%	$EA-CS_{mu}$	0.3145	0.3593	0.3350	0.1413	0.1812	0.1586
	$\text {MOEA/D}$	0.3437	0.5218	0.3588	0.1610	0.2966	0.1767
	$\text {MOEA/D}_{mu}$	0.8038	0.6012	0.6731	0.3853	0.211	0.3275
	$\text {MOEA}\text{- }GO_{FS-PTO}$	0.9051	0.8465	0.8531	0.8820	0.6955	0.7633
	$EA-CS$	0.1387	0.2669	0.1819	0.0942	0.1804	0.1229
50%	$EA-CS_{mu}$	0.2509	0.3097	0.2767	0.1076	0.1442	0.1224
	$\text {MOEA/D}$	0.3005	0.4735	0.3067	0.1406	0.2803	0.1494
	$\text {MOEA/D}_{mu}$	0.7795	0.6009	0.6654	0.3727	0.3641	0.3185
	$\text {MOEA}\text{- }GO_{FS-PTO}$	0.8987	0.8336	0.8412	0.8933	0.7031	0.7663

**Table 11:** Robustness evaluation in terms of *Recall*, *Precision*, and *F*-score. False interactions are added to proteins of maximum number of interactions.

Noise	Algorithm	Yeast-D1			Yeast-D2		
		*Recall*	*Precision*	*F*-score	*Recall*	*Precision*	*F*-score
	$EA-CS$	0.4966	0.5368	0.5154	0.3426	0.3259	0.3335
10%	$EA-CS_{mu}$	0.5957	0.5295	0.5604	0.3236	0.2623	0.2894
	$\text {MOEA/D}$	0.5840	0.5805	0.5652	0.3072	0.2986	0.3462
	$\text {MOEA/D}_{mu}$	0.8218	0.6049	0.6867	0.4427	0.2986	0.3462
	$\text {MOEA}\text{- }GO_{FS-PTO}$	0.9410	0.7499	0.8183	0.8987	0.5584	0.6812
	$EA-CS$	0.3827	0.5027	0.4338	0.2341	0.2890	0.2579
20%	$EA-CS_{mu}$	0.5111	0.4876	0.4988	0.2724	0.2552	0.2632
	$\text {MOEA/D}$	0.5023	0.5891	0.5118	0.2478	0.3066	0.2579
	$\text {MOEA/D}_{mu}$	0.8167	0.6093	0.6834	0.4167	0.3032	0.3398
	$\text {MOEA}\text{- }GO_{FS-PTO}$	0.9308	0.7326	0.8023	0.8960	0.5464	0.6728
	$EA-CS$	0.2853	0.4568	0.3504	0.1619	0.2520	0.1964
30%	$EA-CS_{mu}$	0.4278	0.4598	0.4424	0.2138	0.2317	0.2219
	$\text {MOEA/D}$	0.4238	0.5633	0.4374	0.1940	0.3013	0.2107
	$\text {MOEA/D}_{mu}$	0.8077	0.5802	0.6656	0.4173	0.2899	.03372
	$\text {MOEA}\text{- }GO_{FS-PTO}$	0.9397	0.7228	0.8021	0.8907	0.5380	0.6647
	$EA-CS$	0.2049	0.4070	0.2718	0.1220	0.2334	0.1596
40%	$EA-CS_{mu}$	0.3786	0.4376	0.4055	0.1849	0.2267	0.2031
	$\text {MOEA/D}$	0.3562	0.5255	0.3662	0.1634	0.2944	0.1754
	$\text {MOEA/D}_{mu}$	0.7949	0.5804	0.6565	0.4100	0.2987	0.3323
	$\text {MOEA}\text{- }GO_{FS-PTO}$	0.9397	0.7091	0.7930	0.8973	0.5356	0.6658
	$EA-CS$	0.1539	0.3624	0.2152	0.0900	0.2116	0.1255
50%	$EA-CS_{mu}$	0.3359	0.4257	0.3744	0.1398	0.2072	0.1664
	$\text {MOEA/D}$	0.3250	0.4639	0.3232	0.1542	0.2628	0.1564
	$\text {MOEA/D}_{mu}$	0.8051	0.5683	0.6575	0.4020	0.2915	0.3300
	$\text {MOEA}\text{- }GO_{FS-PTO}$	0.9295	0.7136	0.7970	0.8907	0.5249	0.6548

**Table 12:** Robustness evaluation in terms of *Recall*, *Precision*, and *F*-score. False interactions are added to proteins of minimum number of interactions.

Noise	Algorithm	Yeast-D1			Yeast-D2		
		*Recall*	*Precision*	*F*-score	*Recall*	*Precision*	*F*-score
10%	$EA-CS$	0.5049	0.5371	0.5201	0.3741	0.3396	0.3555
	$EA-CS_{mu}$	0.5846	0.5259	0.5531	0.3380	0.2693	0.2996
	$\text {MOEA/D}$	0.5679	0.5799	0.5571	0.3107	0.2963	0.2963
	$\text {MOEA/D}_{mu}$	0.8192	0.6142	0.6887	0.4207	0.3179	0.3486
	$\text {MOEA}\text{- }GO_{FS-PTO}$	0.9333	0.8577	0.8458	0.8980	0.6561	0.7226
	$EA-CS$	0.3513	0.7216	0.4704	0.3250	0.3292	0.3264
20%	$EA-CS_{mu}$	0.4906	0.4678	0.4785	0.3087	0.2612	0.2828
	$\text {MOEA/D}$	0.4743	0.6031	0.5007	0.2601	0.3333	0.2774
	$\text {MOEA/D}_{mu}$	0.7987	0.6156	0.6881	0.4133	0.3334	0.3489
	$\text {MOEA}\text{- }GO_{FS-PTO}$	0.9269	0.8959	0.8690	0.8907	0.6803	0.7425
	$EA-CS$	0.3233	0.4337	0.3699	0.2609	0.3018	0.2791
30%	$EA-CS_{mu}$	0.4291	0.4376	0.4329	0.2576	0.2408	0.2484
	$\text {MOEA/D}$	0.3864	0.5949	0.4256	0.2173	0.3544	0.2456
	$\text {MOEA/D}_{mu}$	0.7590	0.6004	0.6548	0.3967	0.3219	0.3468
	$\text {MOEA}\text{- }GO_{FS-PTO}$	0.9295	0.8951	0.8847	0.8893	0.7447	0.7739
	$EA-CS$	0.2460	0.3853	0.2995	0.1940	0.2623	0.2221
40%	$EA-CS_{mu}$	0.3816	0.4081	0.3937	0.1971	0.2059	0.2009
	$\text {MOEA/D}$	0.3330	0.5573	0.3648	0.1779	0.3387	0.2016
	$\text {MOEA/D}_{mu}$	0.7590	0.5960	0.6585	0.3967	0.3357	0.3451
	$\text {MOEA}\text{- }GO_{FS-PTO}$	0.9090	0.9314	0.8898	0.8980	0.8219	0.7911
	$EA-CS$	0.1773	0.3241	0.2286	0.1429	0.2256	0.1740
50%	$EA-CS_{mu}$	0.3115	0.3554	0.3314	0.1884	0.2145	0.2001
	$\text {MOEA/D}$	0.2916	0.5153	0.3199	0.1554	0.3306	0.1766
	$\text {MOEA/D}_{mu}$	0.7423	0.5835	0.6318	0.3700	0.3352	0.3336
	$\text {MOEA}\text{- }GO_{FS-PTO}$	0.9064	0.9561	0.8922	0.8800	0.8289	0.7821

**Table 13:** Robustness evaluation in terms of *Recall*, *Precision*, and *F*-score. True interactions are randomly deleted from protein pairs.

Noise	Algorithm	Yeast-D1			Yeast-D2		
		*Recall*	*Precision*	*F*-score	*Recall*	*Precision*	*F*-score
10%	$EA-CS$	0.5189	0.3969	0.4496	0.3181	0.2190	0.2591
	$EA-CS_{mu}$	0.6662	0.5572	0.6065	0.3462	0.2535	0.2925
	$\text {MOEA/D}$	0.6252	0.4682	0.5261	0.3187	0.2048	0.2463
	$\text {MOEA/D}_{mu}$	0.8192	0.5823	0.6645	0.4380	0.3018	0.3394
	$\text {MOEA}\text{- }GO_{FS-PTO}$	0.9410	0.7719	0.8181	0.8987	0.5516	0.6734
	$EA-CS$	0.4195	0.2771	0.3336	0.2717	0.1632	0.2038
20%	$EA-CS_{mu}$	0.6222	0.5421	0.5792	0.3467	0.2543	0.2932
	$\text {MOEA/D}$	0.5579	0.3097	0.3934	0.2881	0.1429	0.1899
	$\text {MOEA/D}_{mu}$	0.8038	0.5979	0.6685	0.4133	0.2818	0.3166
	$\text {MOEA}\text{- }GO_{FS-PTO}$	0.9410	0.7540	0.8106	0.9020	0.5562	0.6718
	$EA-CS$	0.3845	0.2308	0.2883	0.2228	0.1175	0.1537
30%	$EA-CS_{mu}$	0.6226	0.5075	0.5589	0.3622	0.2791	0.3150
	$\text {MOEA/D}$	0.4927	0.2349	0.3163	0.2601	0.1084	0.1527
	$\text {MOEA/D}_{mu}$	0.7769	0.5771	0.6422	0.4453	0.3468	0.3670
	$\text {MOEA}\text{- }GO_{FS-PTO}$	0.9526	0.7526	0.7985	0.9113	0.5593	0.6675
	$EA-CS$	0.2974	0.1622	0.2098	0.1743	0.0842	0.1135
40%	$EA-CS_{mu}$	0.6774	0.5601	0.6130	0.3907	0.293	0.3347
	$\text {MOEA/D}$	0.3964	0.1645	0.2322	0.2247	0.0849	0.1231
	$\text {MOEA/D}_{mu}$	0.7987	0.6199	0.6766	0.4473	0.3651	0.3733
	$\text {MOEA}\text{- }GO_{FS-PTO}$	0.9500	0.7515	0.8002	0.9147	0.5295	0.6556
	$EA-CS$	0.2042	0.0987	0.1330	0.1123	0.0508	0.0698
50%	$EA-CS_{mu}$	0.6615	0.5108	0.5763	0.4080	0.3000	0.3456
	$\text {MOEA/D}$	0.3130	0.1182	0.1715	0.1860	0.0687	0.1002
	$\text {MOEA/D}_{mu}$	0.7513	0.5655	0.6307	0.4513	0.3761	0.3877
	$\text {MOEA}\text{- }GO_{FS-PTO}$	0.9718	0.7512	0.8128	0.9240	0.5353	0.6632

**Table 14:** Robustness evaluation in terms of *Recall*, *Precision*, and *F*-score. True interactions are deleted from proteins of maximum number of interactions.

Noise	Algorithm	Yeast-D1			Yeast-D2		
		*Recall*	*Precision*	*F*-score	*Recall*	*Precision*	*F*-score
10%	$EA-CS$	0.5828	0.5625	0.5721	0.4132	0.3307	0.3671
	$EA-CS_{mu}$	0.6517	0.5549	0.5992	0.3538	0.263	0.3015
	$\text {MOEA/D}$	0.6572	0.5751	0.6031	0.354	0.2884	0.3134
	$\text {MOEA/D}_{mu}$	0.8308	0.6104	0.6848	0.4420	0.3099	0.3493
	$\text {MOEA}\text{- }GO_{FS-PTO}$	0.9397	0.7766	0.8235	0.9027	0.5689	0.6820
	$EA-CS$	0.6085	0.5723	0.5896	0.4198	0.3352	0.3725
20%	$EA-CS_{mu}$	0.6774	0.5572	0.6112	0.3729	0.2665	0.3106
	$\text {MOEA/D}$	0.6717	0.5778	0.6111	0.3572	0.2872	0.3138
	$\text {MOEA/D}_{mu}$	0.8359	0.6049	0.6802	0.4573	0.3136	0.3590
	$\text {MOEA}\text{- }GO_{FS-PTO}$	0.9372	0.7655	0.8186	0.8947	0.5766	0.6897
	$EA-CS$	0.6186	0.5728	0.5945	0.4247	0.3334	0.3733
30%	$EA-CS_{mu}$	0.6756	0.5538	0.6084	0.3838	0.2657	0.3139
	$\text {MOEA/D}$	0.6800	0.5708	0.6106	0.3648	0.2881	0.3176
	$\text {MOEA/D}_{mu}$	0.8359	0.5873	0.6716	0.4513	0.3059	0.3555
	$\text {MOEA}\text{- }GO_{FS-PTO}$	0.9462	0.7774	0.8178	0.8940	0.5612	0.6703
	$EA-CS$	0.6297	0.5714	0.5988	0.4282	0.3326	0.3742
40%	$EA-CS_{mu}$	0.6936	0.5511	0.614	0.3929	0.2672	0.3180
	$\text {MOEA/D}$	0.685	0.5665	0.6099	0.3686	0.286	0.3179
	$\text {MOEA/D}_{mu}$	0.8308	0.5840	0.6672	0.4540	0.3062	0.3512
	$\text {MOEA}\text{- }GO_{FS-PTO}$	0.9423	0.7585	0.8056	0.8960	0.5770	0.6853
	$EA-CS$	0.6364	0.5678	0.5999	0.4263	0.3272	0.3699
50%	$EA-CS_{mu}$	0.6953	0.5497	0.6137	0.3916	0.264	0.3153
	$\text {MOEA/D}$	0.6842	0.5597	0.6055	0.3677	0.2806	0.3139
	$\text {MOEA/D}_{mu}$	0.8179	0.5572	0.6526	0.4580	0.3118	0.3505
	$\text {MOEA}\text{- }GO_{FS-PTO}$	0.9500	0.7632	0.8098	0.9020	0.5610	0.6736

**Table 15:** Robustness evaluation in terms of *Recall*, *Precision*, and *F*-score. True interactions are deleted from proteins of minimum number of interactions.

Noise	Algorithm	Yeast-D1			Yeast-D2		
		*Recall*	*Precision*	*F*-score	*Recall*	*Precision*	*F*-score
10%	$EA-CS$	0.4692	0.3194	0.3799	0.2721	0.1613	0.2024
	$EA-CS_{mu}$	0.6444	0.5307	0.5819	0.3544	0.2649	0.303
	$\text {MOEA/D}$	0.5951	0.4259	0.488	0.3053	0.1661	0.2129
	$\text {MOEA/D}_{mu}$	0.8051	0.5938	0.6755	0.4220	0.3123	0.3491
	$\text {MOEA}\text{- }GO_{FS-PTO}$	0.9269	0.7779	0.8248	0.9067	0.5688	0.6827
	$EA-CS$	0.4581	0.2999	0.3623	0.2724	0.1537	0.1964
20%	$EA-CS_{mu}$	0.6581	0.5232	0.5827	0.3622	0.2586	0.3016
	$\text {MOEA/D}$	0.5737	0.3984	0.462	0.2941	0.1615	0.2063
	$\text {MOEA/D}_{mu}$	0.8372	0.5892	0.677	0.4227	0.3063	0.3425
	$\text {MOEA}\text{- }GO_{FS-PTO}$	0.9333	0.7709	0.8192	0.8953	0.5722	0.6859
	$EA-CS$	0.4695	0.3035	0.3685	0.2695	0.1507	0.1932
30%	$EA-CS_{mu}$	0.6385	0.4849	0.5508	0.366	0.2494	0.2965
	$\text {MOEA/D}$	0.5763	0.3729	0.4453	0.2761	0.1461	0.189
	$\text {MOEA/D}_{mu}$	0.7936	0.5448	0.6310	0.4240	0.3070	0.3419
	$\text {MOEA}\text{- }GO_{FS-PTO}$	0.9474	0.7601	0.8184	0.9027	0.5596	0.6804
	$EA-CS$	0.4324	0.2589	0.3238	0.2597	0.1284	0.1717
40%	$EA-CS_{mu}$	0.5872	0.395	0.472	0.3751	0.2354	0.2892
	$\text {MOEA/D}$	0.5264	0.2998	0.3763	0.2822	0.1282	0.1752
	$\text {MOEA/D}_{mu}$	0.7244	0.4379	0.5380	0.4080	0.2705	0.3121
	$\text {MOEA}\text{- }GO_{FS-PTO}$	0.9359	0.7668	0.8066	0.9020	0.5589	0.6757
	$EA-CS$	0.2699	0.1341	0.1791	0.1852	0.0907	0.1218
50%	$EA-CS_{mu}$	0.5316	0.3535	0.4245	0.3489	0.2577	0.2964
	$\text {MOEA/D}$	0.384	0.1544	0.2194	0.248	0.0991	0.1414
	$\text {MOEA/D}_{mu}$	0.6103	0.3930	0.4706	0.3920	0.3221	0.3374
	$\text {MOEA}\text{- }GO_{FS-PTO}$	0.9346	0.7702	0.8122	0.9093	0.5536	0.6807

## Conclusions

This study has successfully redefined the challenge of detecting protein complexes in PPI networks by framing it as a multi-objective optimization problem. A new model for complex detection is introduced, distinguished by integrating a heuristic perturbation operator. The incorporation of GO heuristic operators has proven crucial for boosting the performance of evolutionary algorithms, yet this approach has not been extensively explored in existing research. Our proposed GO-based heuristic operators, which leverage functional similarity among gene products, have demonstrated significant effectiveness in detecting complexes within PPI networks. These findings underscore the growing viability of GO-based methods in addressing real-world biological problems. However, to fully assess the potential of these evolutionary algorithms, future research should explore additional algorithm characteristics and PPI network properties, including complex overlapping and varying complex sizes, through more extensive experimental investigations.

## Data Availability

The datasets used in this study are available in the supplementary files.
